# Selective Desulfurization Significantly Expands Sequence Variety of 3′-Peptidyl–tRNA Mimics Obtained by Native Chemical Ligation

**DOI:** 10.1002/cbic.201200368

**Published:** 2012-07-11

**Authors:** Anna-Skrollan Geiermann, Ronald Micura

**Affiliations:** [a] Institute of Organic Chemistry, CCB: Center for Chemistry and Biomedicine, University of Innsbruck6020 Innsbruck (Austria) E-mail: ronald.micura@uibk.ac.at

**Keywords:** bioconjugates, nucleoside modification, peptides, RNA solid-phase synthesis

RNA–peptide conjugates that mimic acylated tRNA termini are valuable compounds for structural and functional studies of the ribosomal elongation cycle, particularly if they contain a hydrolysis-resistant linkage between the RNA and the peptide moiety.^1^ In a very reduced form, puromycin ((*S*)-3′-((2-amino-3-(4-methoxyphenyl)-1-oxopropyl)amino)-3′-desoxy-*N*,*N*-dimethyladenosine), which possesses an amide instead of an ester junction, represents such a stable conjugate.^2^ This mimic, for example, was positioned as a substrate in the P-site of the ribosomal peptidyl transferase center (PTC) to capture snapshots along the route to peptide bond formation, as analyzed by X-ray crystallography.^3^ A more recent example made use of short, stable, 3′-aminoacyl-RNA conjugates to explore how the nascent peptide chain triggers ribosomal stalling, as analyzed by a variety of biochemical methods.^4^ Straightforward experimental approaches to synthesize this type of bioconjugate are expected to stimulate further investigations and functional characterization of the different states along the ribosomal elongation cycle.^5^

The total syntheses of 3′-aminoacyl- and 3′-peptidyl-RNA represent substantive challenges for organic chemists;2c,d therefore, a central focus of our research is the de novo synthesis of these derivatives.^6^ We have recently elaborated a convergent strategy that involves native chemical ligation (NCL) of 3′-cysteinylamino-3′-deoxy-RNA and peptide thioesters.^7^ NCL was originally developed to link unprotected peptide fragments under mild conditions, and this approach eventually emerged as a major advance in chemical protein synthesis.^8^ Our work has shown that NCL can also work efficiently in the context of RNA^7^ and therefore might serve as a launching point for further investigations in the field of RNA bioconjugation.

Within this framework, we considered here an important extension of the NCL-based strategy towards 3′-peptidyl-tRNA mimics, specifically their desulfurization (Scheme 1). As NCL in its original version is cysteine dependent, considerable efforts have been expended toward the development of cysteine-free ligation methods,^9^ while others aimed to convert the erstwhile cysteine into an alanine to circumvent the requirement for cysteine in the proposed ligation site.^10^ Soon after the pioneering work by Yan and Dawson,10a who achieved this conversion through the use of Raney nickel or Pd/Al_2_O_3_, a metal-free version for cysteine thiol reduction was reported by Danishefsky and co-workers.^11^ Further important contributions have constituted efficient protocols for desulfurization of a γ-thiol valine by the same group^12^ and for penicillamine by Seitz and co-workers; ^13]^ both promote valine—a rather abundant amino acid (6.6 % as opposed to 1.7 % frequency for cysteine)—as a practicable ligation site.

**Scheme 1:**
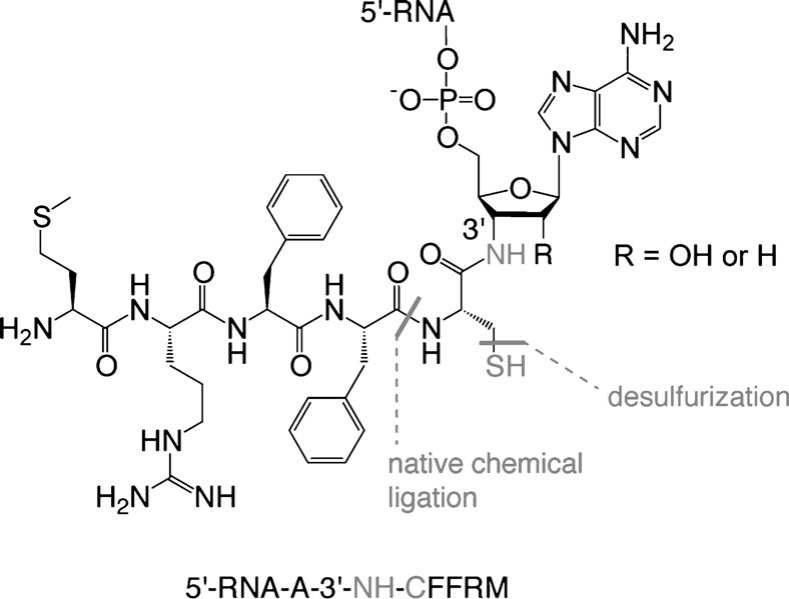
Synthetic strategy and structure of an exemplary amide-linked 3′-peptidyl-tRNA mimic. Sequence annotation of conjugate (bottom): note that peptide is annotated from C to N terminus.

Involving RNA–peptide conjugates in metal- or radical-based desulfurization appears difficult at first sight, as RNA is rather fragile under the required conditions. Many hurdles are to be expected, such as irreversible adsorption of the nucleic acid on the metal surface or unspecific phosphodiester cleavage catalyzed by the metal ions involved.^14^ Moreover, radical chemistry with RNA is associated with nucleic acid damage in general^15^ and also with “hydroxyl radical footprinting” (a method that exploits hydroxyl radicals to promote the cleavage of RNA at nucleotide resolution),^16^ thus clearly reflecting the unpleasant potential of the planned undertaking. However, other examples exist, such as copper(I)-catalyzed click chemistry, for which the reaction conditions were allegedly incompatible with RNA; yet, after adjustments to the method, RNA is now successfully utilized for this important bioconjugation method.^17^

To begin our endeavors, we synthesized a series of 3′-cysteinylamino-3′-deoxy-RNAs and short peptide 4-(*N*-(2-aminoethyl)carbamoyl)benzylthioesters; the latter are referred to as ABT thioesters (Scheme 2).^18^ NCL was conducted under the optimized conditions based on our previously elaborated protocol,^7^ involving concentrations of 0.25 mm cysteinyl-RNA and 8 mm peptide thioester in Tris buffer (1 m) at pH 8.0, in the presence of urea (7 m) and tris(carboxyethyl)phosphine (TCEP; 0.1 m) for *S*-*t*Bu removal in situ,^19^ and thiophenol (2 % (*v*/*v*)) for formation of more reactive thioesters.^20^ After a typical reaction time of 20 h at 25 °C, the reaction mixture was applied to centrifugal concentrators with ultrafiltration membranes for desalting and separation from excess peptide thioesters; finally, the crude products were lyophilized.

**Scheme 2:**
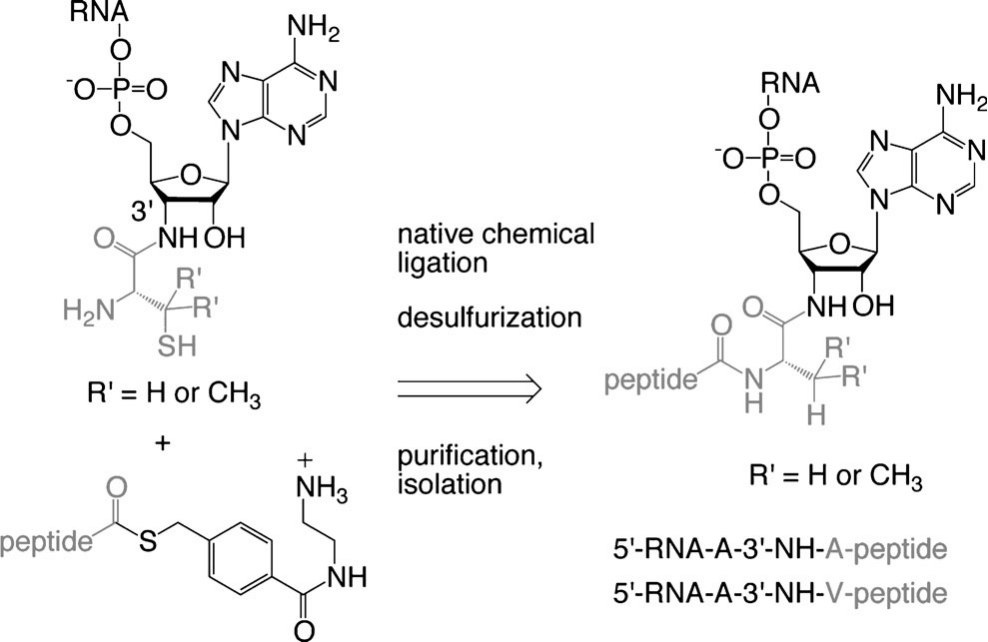
Workflow for NCL–desulfurization procedure to generate 3′-peptidyl-tRNA mimics.

At this point, the actual desufurization reaction of cysteine was started. Without further purification, the residue was exposed to tris(carboxyethyl)phosphine (TCEP) in combination with a water-soluble radical initiator (2,2′-azobis(2-methylpropionamidine)dihydrochloride, V-50), comparable to the protocol for cysteine reduction in peptides by Wan and Danishefsky.^11^ From a mechanistic point of view, the abstraction of a hydrogen atom from the thiol group results in its reduction by TCEP to form an alanyl radical which receives a hydrogen from a suitable additive. We chose glutathione instead of EtSH or *t*BuSH, being the more powerful hydrogen donor as recommended by Seitz and co-workers.^13^ The reactions performed here were optimal at conjugate concentrations of 0.6 mm in 240 mm aqueous sodium phosphate buffer adjusted to pH 7.5, containing 200 mm TCEP, 16 mm V-50, and 4 mm glutathione, for 6 to 12 h at 37 °C.

Figure 1 illustrates typical NCL–desulfurization reactions of RNA–peptide conjugates, analyzed by HPLC and LC–ESI mass spectrometry. The overall yields of the two-step procedure were determined from HPLC profiles and ranged from 55 to 75 % with respect to 3′-cysteinyl-RNA (Table 1, conjugates **1** to **7**). The major byproduct was isolated, characterized by mass spectrometry, and assigned as desulfurized starting material, namely, 3′-alanylamino-3′-deoxy-RNA. This side product is most likely generated from unreacted 3′-cysteinyl-RNA; however, it might also form earlier during side chain deprotection of Cys(*S*-*t*Bu)^21^ using TCEP as the commonly accepted and most effective starter for NCL reactions. Although this reagent could limit the overall ligation–desulfurization yields by converting a minor amount of starting material into unreactive 3′-alanyl species, alternatives for *S*-*t*Bu deprotection (e.g., DTT) were at much higher cost with respect to overall yields of the isolated target conjugates.

**Figure 1:**
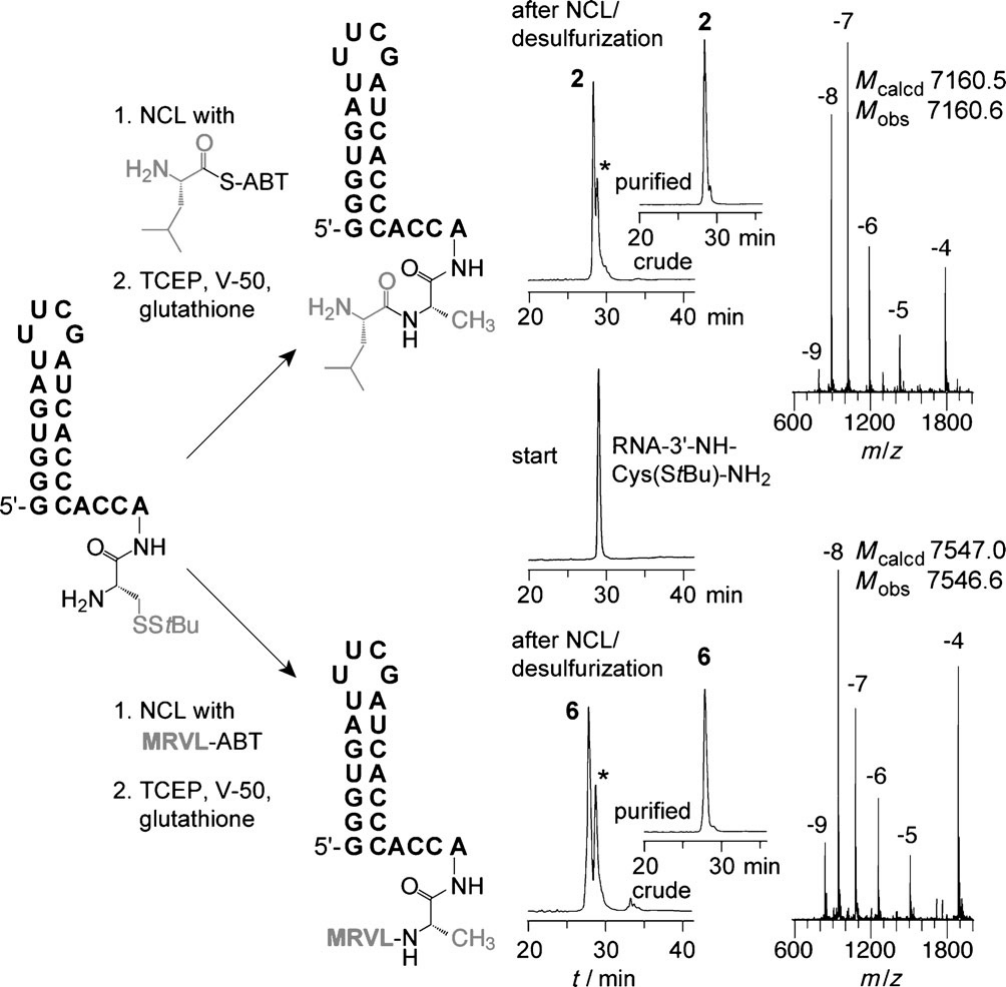
NCL–desulfurization of 3′-peptidylamino-3′-deoxy-RNA. Anion-exchange HPLC traces and LC–ESI mass spectra of 5′-RNA-3′-NH-AL (top) and 5′-RNA-3′-NH-ALVRM (bottom); Reaction conditions: NCL: *c*_RNA_=0.25 mm, *c*_peptide−ABT_=8 mm; 0.1 m TCEP, 2 % (*v*/*v*) PhSH, 1 m Tris⋅HCl, pH 8, 20 h, 25 °C; desulfurization: *c*_conjugate_=0.6 mm; 200 mm TCEP, 16 mm V-50, 4 mm glutathione, 240 mm sodium phosphate, pH 7.5, 12 h, 37 °C. An asterisk indicates a byproduct that was identified as desulfurized starting material (Supporting Information).

**Table 1:** 3′-Peptidyl-tRNA-mimics obtained by NCL–desulfurization

No	Sequence	Yield [%]	*M*_Wcalcd_[amu]	*M*_Wobs_[amu]
1	5′-p-CUCCA-3′-NH-AFFRM	65	2220.7	2220.6
2	5′-G_3_UGAU_3_CGAUCAC_3_ACCA-3′-NH-AL	75	7160.5	7160.6
3	5′-G_3_UGAU_3_CGAUCAC_3_AC_2_A-3′-NH-AGFFM	70	7529.9	7529.7
4	5′-G_3_UGAU_3_CGAUCAC_3_AC_2_A-3′-NH-AFFRM	55	7629.1	7629.1
5	5′-G_3_UGAU_3_CGAUCAC_3_AC_2_A-3′-NH-ATLLM	65	7506.0	7505.6
6	5′-G_3_UGAU_3_CGAUCAC_3_AC_2_A-3′-NH-ALVRM-NH_2_	65	7547.0	7546.6
7	5′-G_3_UGAU_3_CGAUCAC_3_AC_2_A-3′-NH-AWVRM	70	7620.1	7619.9
8	5′-G_3_UGAU_3_CGAUCAC_3_AC_2_A-3′-NH-VGFFM	20	7558.0	7557.6
9	5′-d(CTCCGGAACGCGCCTCC)dA-3′-NH-ALVRM	65	5975.3	5975.1
10	5′-d(CTCCGGAACGCGCCTCC)dA-3′-NH-GALVRM	55	6032.3	6032.6

RNA is annotated in the 5′ to 3′ direction; peptide from the C to N terminus.

Table 1 lists a series of 3′-peptidyl-RNAs (**1** to **7**) with Xaa-Ala (Xaa=any amino acid) ligation sites, synthesized by the approach presented here. To define the limits, we also examined the principal accessibility of Xaa-Val sites in 3′-peptidyl-tRNA mimics. We decided in favor of β,β-dimethylcysteine (penicillamine, Pen)^13^ rather than γ-thiol valine as a precursor^12^ because of its commercial availability with various protection patterns. We consequently synthesized the novel 3′-(β,β-dimethylcysteinyl)amino-3′-deoxyadenosine-modified solid support (Supporting Information) for RNA solid-phase synthesis, on the basis of a previously elaborated route for generating 3′-aminoacylamino-3′-deoxyadenosine derivatives.6a After having prepared 3′-penicillaminyl-RNA and the corresponding peptide ABT ester, the ligation–desulfurization protocol developed herein furnished conjugate 5′-G_3_UGAU_3_CGAUCAC_3_AC_2_A-3′-NH-VGFFM-NH_2_ **8** in low but acceptable yield, leaving room for further optimization; unreacted and desulfurized starting material were the major byproducts. Another issue we wanted to test was the compatibility of the elaborated desulfurization conditions with DNA, which might be more sensitive than RNA towards free radicals and could therefore be harmed.^14^, 15a We therefore synthesized and incubated a 3′-cysteinyl-DNA precursor with the corresponding peptide thioester to form conjugate **9** and were pleased to observe good yields. We confirmed this extension to DNA for a second 3′-peptidyl-DNA conjugate (**10**) with an internal alanine ligation site.

Many of the conjugates synthesized here represent tRNA acceptor stem–loop structures carrying an arginine-containing short peptide. Such conjugates have resisted direct access by solid-phase synthesis in which both peptide and RNA are assembled on the same functionalized solid support, followed by cleavage and deprotection of the whole conjugate.6a, d We furthermore underline that the conditions elaborated here for desulfurization are compatible with methionine, which carries a thioether moiety that turned out to be sufficiently stable. Moreover, the pentapeptide sequences targeted here relate to macrolide antibiotic resistance peptides.^22^ When these peptides are translated, they can render the ribosome resistant to macrolide antibiotics by a mechanism yet to be explored.

We note that we focused on free-radical-mediated methods for desulfurization from the beginning of our investigation as an early report by Hecht and co-workers on the desulfurization of thio-modified nucleosides in tRNA indicated severe limitations for metal-based approaches.^23^ Likewise, desulfurization under oxidative conditions was excluded from our studies. Although such conditions would be compatible with the RNA strand (e.g., see oxidative desulfurization of 2-thiouridines in oligonucleotides),^24^ they are not compatible with the peptide moiety owing to elimination reactions (generating dehydroalanine) or the concomitant oxidation of methionine residues.

In summary, we have demonstrated a convenient synthetic strategy towards hydrolysis-resistant 3′-peptidyl-tRNA mimics. By relying on NCL and subsequent desulfurization, sequences that are inaccessible by other methods (including NCL alone) become possible for this important class of RNA conjugates.

## Experimental Section

**Desulfurization procedure:** After native chemical ligation of a 3′-peptidyl-RNA conjugate, which was carried out as described previously,^7^ the reaction mixture (∼12 μL) was diluted with nanofiltered water (500 μL), transferred into a centrifugal concentrator with an ultrafiltration membrane (Vivaspin 500, 3000 MWCO PES, product number VS0191), and centrifuged for 20 min (Eppendorf MiniSpin, 13 400 rpm). The solution in the lower reservoir was discarded. Then, 500 μL of 100 mm ammonium citrate solution was added to the upper reservoir, followed by centrifugation for another 20 min. After a final washing step with H_2_O (500 μL), the upper solution containing the crude NCL product was lyophilized to dryness and subsequently dissolved in freshly prepared, degassed desulfurization reagent stock solutions. Final concentrations were: 0.6 mm conjugate, 240 mm sodium phosphate (pH 7.5), 200 mm TCEP, 16 mm V-50, and 4 mm glutathione. The reaction mixture was sonicated for 10 s and incubated under argon atmosphere for 6 to 12 h at 37 °C. Analysis of the reaction was performed by direct injection of an aliquot onto an anion-exchange chromatographic column (Dionex DNA-Pac PA100). Purification was also performed via anion-exchange chromatography, followed by desalting on a C18 SepPakPlus cartridge (Waters) and lyophilization. All conjugates were analyzed by LC–ESI mass spectrometry to confirm their expected molecular weights (see Supporting the Information for details).

## Supplementaary material

Detailed facts of importance to specialist readers are published as ”Supporting Information”. Such documents are peer-reviewed, but not copy-edited or typeset. They are made available as submitted by the authors.
