# Supplementary material for: Selective Desulfurization Significantly Expands Sequence Variety of 3′-Peptidyl–tRNA Mimics Obtained by Native Chemical Ligation
Source: Chembiochem. 2012 Jul 11;13(12):1742–5. doi: 10.1002/cbic.201200368 (PMC3430856; doi:10.1002/cbic.201200368)
Supplement: Supplementary file 1 [file cbic0013-1742-SD1.pdf]

CHEMBIOCHEM

Supporting Information

© Copyright Wiley-VCH Verlag GmbH & Co. KGaA, 69451 Weinheim, 2012

Selective Desulfurization Significantly Expands Sequence Variety of 3′-Peptidyl–tRNA Mimics Obtained by Native Chemical Ligation

Anna-Skrollan Geiermann and Ronald Micura*[a]

cbic_201200368_sm_miscellaneous_information.pdf

Content:

Supporting Methods

1 General S3

1.1 General information for building block synthesis S3

1.2 General information for oligonucleotide synthesis S3

1.3 General information for peptide synthesis S3

2 Synthesis of *ß,ß*-dimethylcysteine (penicillamine, Pen) functionalized rA$^{3'NH}$ solid support rA-Pen·3 S4

3 Synthesis of cysteine functionalized dA$^{3'NH}$ solid support dA-GlyCys S11

4 Solid phase synthesis, deprotection, and purification of 3'-aminoacyl-oligonucleotides S18

4.1 Deprotection of the 5'-*O*-tert.-butyldimethylsilyl (tbdms) group of solid supports dA-Cys·4 and dA-GlyCys prior to DNA synthesis S18

4.2 Oligonucleotide solid phase synthesis on 3'-aminoacyl-functionalized solid supports S18

4.3 Deprotection of the *N*-9-(fluorenyl)methoxycarbonyl (Fmoc) group after RNA synthesis on solid support rA-Pen·3 S19

4.4 Deprotection of the *N*-allyloxycarbonyl (alloc) group after oligonucleotide synthesis on solid supports rA-Cys·3, dA-Cys·4, and dA-GlyCys S19

4.5 Deprotection and cleavage of RNA-Pen(NPys) conjugates S19

4.6 Deprotection and cleavage of a 5 nt RNA-Cys(S*t*Bu) conjugate S19

4.7 Deprotection and cleavage of DNA-Cys(S*t*Bu) conjugates S20

4.8 Purification of 3'-aminoacyl-oligonucleotides S20

4.9 Mass spectrometry of 3'-aminoacyl-oligonucleotides S20

5 Preparation of amino-modified peptide thioester MRFF-ABT S20

5.1 Solid phase peptide synthesis S20

5.2 Synthesis of peptide thioester MRFF-ABT S21

5.3 Analysis and purification of peptides S21

5.4 Mass spectrometry of peptides S21

6 Desulfurization of 3'-peptidyl-oligonucleotides after native chemical ligation S21

6.1 Analysis and purification of 3'-peptidyl-oligonucleotides after native chemical ligation/desulfurization S22

6.2 Mass Spectrometry of 3'-peptidyl-oligonucleotides S22

Supporting Figures

Supporting Figure 1 S23
Supporting Figure 2 S24
Supporting Figure 3 S25
Supporting Figure 4 S26
Supporting Figure 5 S27
Supporting Figure 6 S28
Supporting Figure 7 S29

References S30

1 General

1.1 General information for building block synthesis

^{1}H and ^{13}C NMR spectra were recorded on a *Bruker* DRX 300 MHz or *Bruker* UltraShield™ 600 MHz. The chemical shifts are reported relative to TMS in ppm and referenced to the residual proton signal of the deuterated solvent: $CDCl_3$ (7.26 ppm) and DMSO (2.50 ppm) for ^{1}H NMR spectra. Referencing for ^{13}C NMR spectra: $CDCl_3$ (77.1 ppm) and DMSO (39.5 ppm). ^{1}H and ^{13}C assignments were based on COSY and HSQC experiments. Coupling constant *J* is reported in Hz. MS experiments were performed on a Finnigan LCQ Advantage MAX ion trap instrumentation (*Thermo Fisher Scientific*), samples were solubilized in MeOH/H_2O (1/1) and analyzed in the positive-ion mode. Conditions for flow injections were MeOH/H_2O (1/1) with a flow rate of 100 µL/min. Reaction control was performed with analytical thin-layer chromatography (TLC, *Machery-Nagel*) on silica plates with fluorescent indicator. Flash column chromatography was carried out on silica gel 60 (70-230 mesh). Chemical reagents and solvents were purchased from commercial suppliers (*Sigma-Aldrich*, *Acros*) and used without further purification. Organic solvents for reactions were dried overnight over freshly activated molecular sieves (4Å). 9-(3'-Amino-2',3'-dideoxy-*ß*-D-ribofuranosyl)adenine and 9-(*ß*-D-arabinofuranosyl)adenine were purchased from *Metkinen Chemistry*. 3'-Amino-6-*N*-[(di-n-butylamino)methylene]-3'-deoxy-5'-*O*-(4,4'-dimethoxytrityl)-*ß*-D-adenosine was synthesized in seven steps according to the literature.[1,2,3,4] Cysteine functionalized solid supports dA-Cys·4 and rA-Cys·3 for solid phase oligonucleotide synthesis were prepared as described in reference 4. Boc-Pen(NPys)-OH was purchased from *Bachem*.

1.2 General information for oligonucleotide synthesis

RNA and DNA standard nucleoside phosphoramidite building blocks and solid supports were purchased from *GlenResearch*, *ChemGenes* and *GE Healthcare*. Other reagents and solvents were purchased from commercial suppliers (*Sigma-Aldrich*, *Acros*) and used without further purification. Organic solvents were dried overnight over freshly activated molecular sieves (4Å). For all reactions and purifications nanofiltered water was used. RNA-Cys(StBu) conjugates 21nt rA-3'-NH-Cys(StBu), 21nt dA-3'-NH-Cys(StBu) and 17nt rA-3'-NH-Cys(StBu) were prepared as described in reference 4.

1.3 General information for peptide synthesis

Fmoc-protected L-amino acids were purchased from *Iris Biotech*, *Bachem* or *Sigma Aldrich*. Resins for solid phase peptide synthesis were purchased from *Novabiochem and Iris Biotech*. Hydroxybenzotriazole monohydrate (HOBt) and *O*-(benzotriazol-1-yl)-*N*,*N*,*N*',*N*'-tetramethyluronium hexafluorophosphate (HBTU) were purchased from *Bachem*. *O*-(7-azabenzotriazol-1-yl)-*N,N,N',N'*-tetramethyluronium hexafluorophosphate (HATU) was purchased from Sigma-Aldrich. Dimethylformamide (DMF) for peptide synthesis was purchased from *Biosolve*. Peptide thioesters Leu-ABT, MFFG-ABT, MLLT-ABT, MRVL-ABT and MRVW-ABT were synthesized as described in reference 4.

2 Synthesis of *ß,ß*-dimethylcysteine (penicillamine, Pen) functionalized rA$^{3'NH}$ solid support rA-Pen·3

S-(3-nitro-2-pyridine-sulfenyl)-*ß,ß*-dimethylcysteine (H-Pen(NPys)-OH)

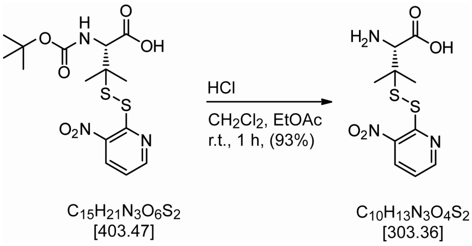

Boc-Pen(NPys)-OH (500 mg, 1.24 mmol) was dissolved in 2.5 mL CH_2Cl_2 and 5 mL 4 M HCl in ethylacetate (EtOAc) and stirred for 1 hour at roomtemperature. After evaporation of the solvents the reisdue was taken up in H_2O and washed three times with diethylether. The aqueous phase is evaporated to dryness and the product is obtained as yellow solid.

Yield: 349 mg (1.15 mmol, 93%). ^{1}H NMR (300 MHz, DMSO): δ 1.46 (s, 3 H, H_3C-C(ß)); 1.54 (s, 3 H, H_3C-C(ß)); 3.76 (s, 1 H, H-C(α)); 7.68 (dd, ^3J = 8.2, 1 H, H-C(ar)); 8.69 (d, ^3J = 8.2, 1 H, H-C(ar)); 8.96 (bs, 1 H, HN); 9.03 (d, ^3J = 4.3, 1 H, H-C(ar)); 14.07 (bs, 1 H, HOOC). ^{13}C NMR (300 MHz, DMSO): δ 23.27 (C(CH_3)); 27.27 (C(CH_3)); 52.57 ($C(CH_3)_2$); 60.34 (C(α)); 123.61 (C(ar)); 135.95 (C(ar)); 143.75; 155.24 (C(ar)); 168.78.

^{1}H NMR (300 MHz, DMSO):

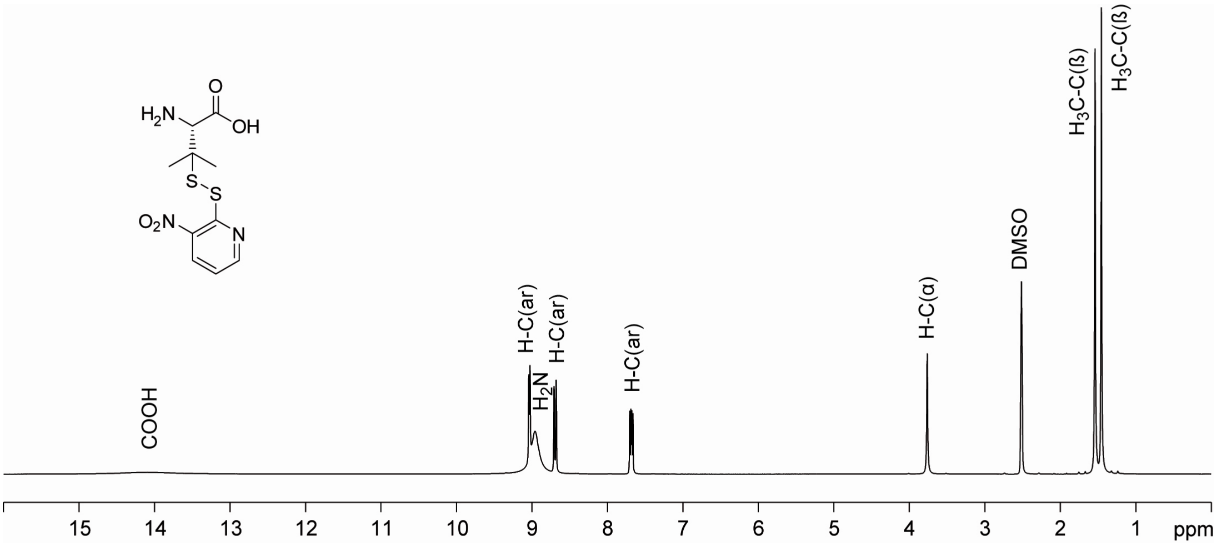

^{13}C NMR (300 MHz, DMSO):

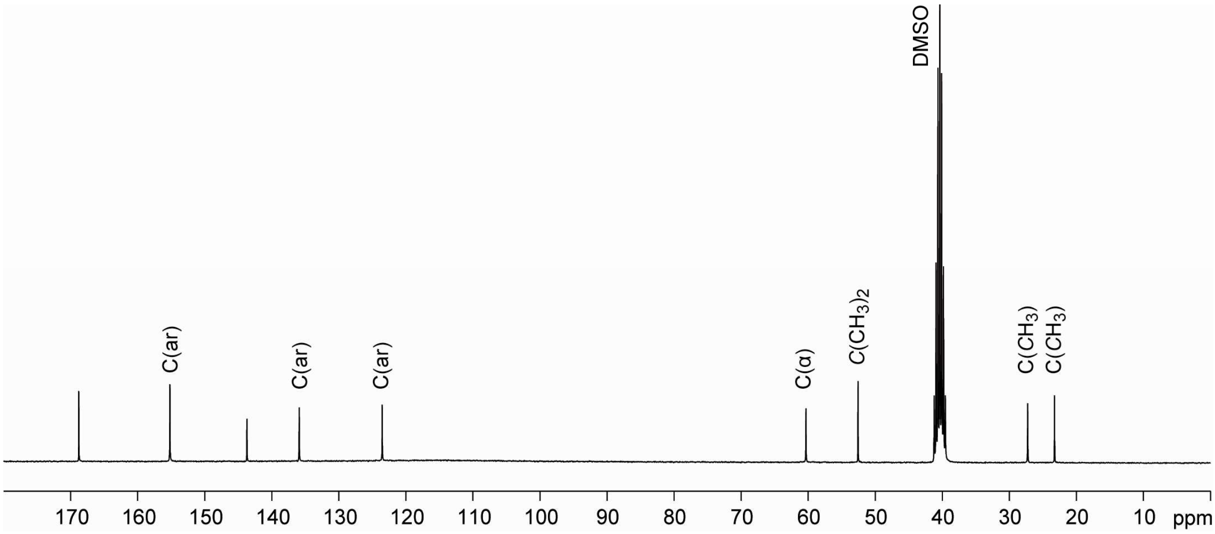

***N*-(9-fluorenyl)methoxycarbonyl-*S*-(3-nitro-2-pyridine-sulfenyl)-*ß*,*ß*-dimethylcysteine (Fmoc-Pen(NPys)-OH)**

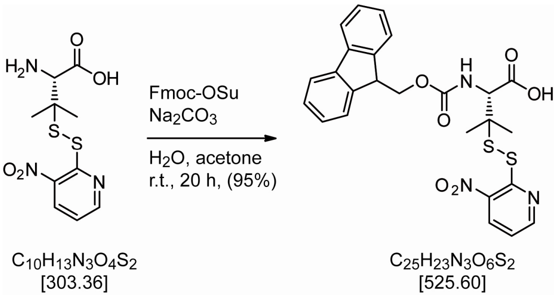

H-Pen(NPys)-OH (349 mg, 1.15 mmol), 1.1 eq *N*-(9-fluorenylmethoxycarbonyloxy)succinimide (Fmoc-OSu, 427 mg, 1.27 mmol) and 1.1 eq Na_2CO_3 (135 mg, 1.27 mmol) are dissolved in 15 mL H_2O/acetone (1:1). At pH 9 the reaction was stirred for 16 hours at room temperature. Then ethylacetate was added and the reaction mixture was acidified with concentrated HCl to pH 1. the organic phase was washed three times with H_2O and ried over Na_2SO_4. The product was purified by column chromatography on SiO_2 (CH_2Cl_2/MeOH, 100/0 - 96/4 v/v) and obtained as yellow solid.

Yield: 574 mg (1.09 mmol, 95%). TLC (CH_2Cl_2/MeOH, 8/2 v/v): R_f = 0.46. ^1H NMR (300 MHz, $CDCl_3$): δ 1.41 (s, 3 H, H_3C-C(ß)); 1.61 (s, 3 H, H_3C-C(ß)); 4.18 (t, ^3J = 6.3, 1 H, H(a)C-O (Fmoc)); 4.41 – 4.47 (m, 3 H, H-C(α, Pen), H(b)C-O (Fmoc) and H-C(9, Fmoc)); 6.92 (bs, 1 H, NH); 7.23 – 7.42 (m, 5 H, H-C(ar)); 7.58 (t, ^3J = 5.9, 2 H, H-C(ar)); 7.74 (d, ^3J = 7.1, 2 H, H-C(ar)); 8.49 (m, 2 H, H-C(ar)); 12.26 (bs, 1 H, HOOC). ^{13}C NMR (300 MHz, $CDCl_3$): δ 24.38 (C(CH$_3$)); 26.50 (C(CH$_3$)); 46.93 (C (9, Fmoc)); 52.55 (C(CH$_3$)$_2$); 61.44 (C(α)); 66.72 (O-CH_2, Fmoc); 119.84 (C(ar)); 121.62 (C(ar)); 124.83 (C(ar)); 126.96 (C(ar)), 127.59 (C(ar)), 134.70 (C(ar)); 141.01; 142.79; 143.31; 143.54; 152.71 (C(ar)); 156.16; 157.36; 171.93.

^{1}H NMR (300 MHz, $CDCl_3$):

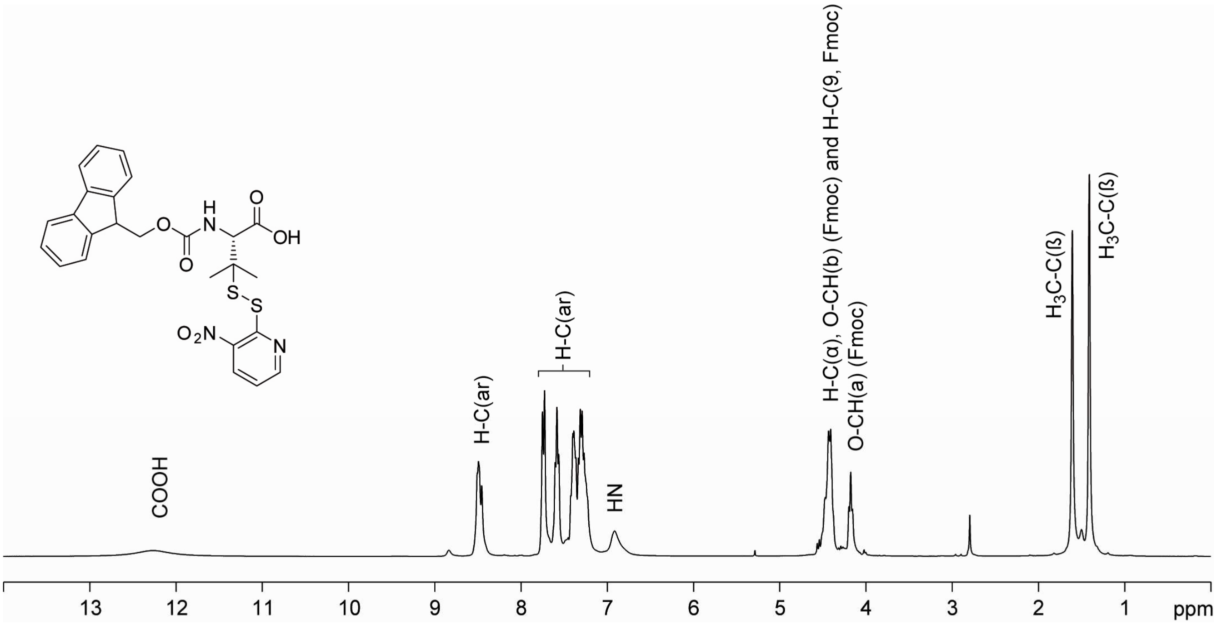

^{13}C NMR (300 MHz, $CDCl_3$):

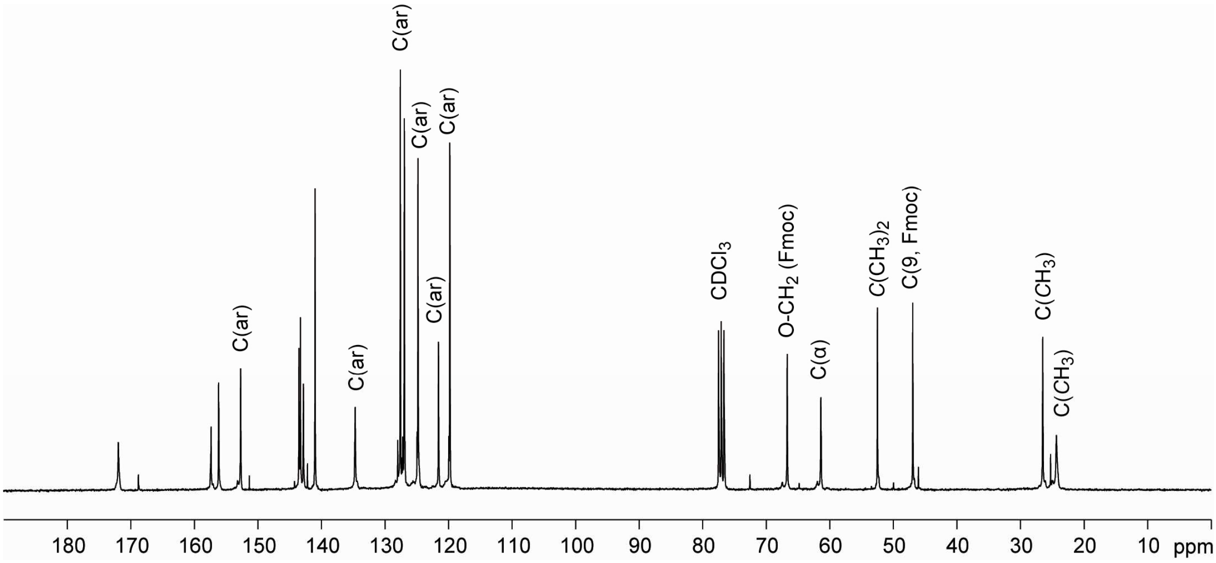

6-*N*-[(di-*n*-butylamino)methylene]-3'-{[*N*-(9-fluorenyl)methoxycarbonyl-*S*-(3-nitro-2-pyridine-sulfenyl)-L-*ß*,*ß*-dimethylcysteinyl]amino}-3'-deoxy-5'-*O*-(4,4'-dimethoxytrityl)-*ß*-D-adenosine (rA-Pen·1)

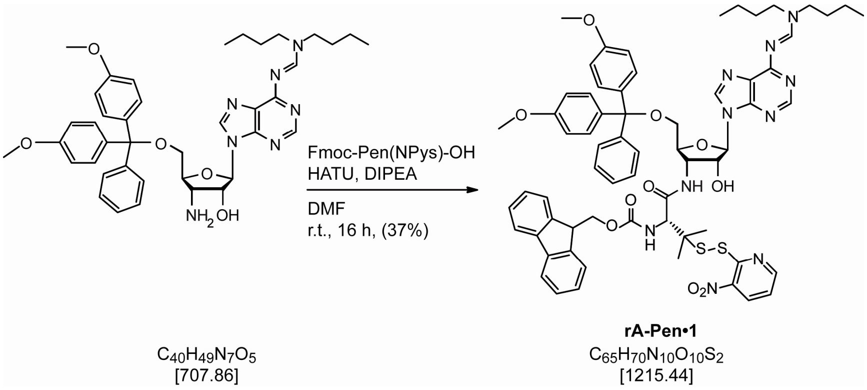

Fmoc-Pen(NPys)-OH (66 mg, 0.26 mmol) was activated with 2-(7-Aza-1H-benzotriazole-1-yl)-1,1,3,3-tetramethyluronium hexafluorophosphate (HATU, 99 mg, 0.26 mmol) and *N*,*N*-diisopropylethylamine (DIPEA, 44 µL) in 2.7 mL dry DMF under argon atmosphere for 8 minutes. Then 3'-Amino-6-*N*-[(di-*n*-butylamino)methylene]-3'-deoxy-5'-*O*-(4,4'-dimethoxytrityl)-*ß*-D-adenosine (141 mg, 0.20 mmol) in 1.3 mL dry DMF were added and stirred for 16 hours at room temperature under argon atmosphere. After evaporation of the solvent, the crude product was dissolved in CH_2Cl_2 and washed with 5% citric acid, saturated $NaHCO_3$ solution and saturated NaCl solution. After drying the organic phase over Na_2SO_4 the product was purified by column chromatography on SiO_2 (CH_2Cl_2/MeOH, 98/2 v/v). The product was obtained as yellow foam.

Yield: 90 mg (0.07 mmol, 37%). TLC (CH_2Cl_2/MeOH, 93/7 v/v): R_f = 0.54. ^{1}H NMR (300 MHz, $CDCl_3$): δ 0.91 – 0.97 (m, 6 H, $N(CH_2CH_2CH_2CH_3)_2$); 1.14 (s, 3 H, H_3C-C(ß, Pen)); 1.30 – 1.43 (m, 4 H, $N(CH_2CH_2CH_2CH_3)_2$); 1.46 (s, 3 H, H_3C-C(ß, Pen)); 1.59 – 1.71 (m, 4 H, $N(CH_2CH_2CH_2CH_3)_2$); 3.41 (m, 3 H, H(a)-C(5') and $N(CH_2CH_2CH_2CH_3)$); 3.54 (m, 1 H, H(b)-C(5')); 3.65 – 3.76 (m, 8 H, 2 × O-CH_3 (DMT) and $N(CH_2CH_2CH_2CH_3)$); 4.15 (m, 1 H, H(a)C-O (Fmoc)); 4.33 – 4.46 (m, 3 H, H-C(α, Pen), H(b)C-O (Fmoc) and H-C(9, Fmoc)); 4.55 (m, 1 H, H-C(4')); 4.70 (m, 1 H, H-C(3')); 4.92 (m, 1 H, H-C(2')); 6.10 (s, 1 H, H-C(1')); 6.74 (d, 5 H, ^3J = 8.5, 4 H, H-C(ar) and HN (Pen)); 7.15 – 7.38 (m, 16 H, H-C(ar)); 7.53 (m, 2 H, H-C(ar)); 7.72 (d, ^3J = 8.0, 2 H, H-C(ar)); 8.17 (s, 1 H, H-C(8)); 8.49 (bs, 2 H, H-C(2) and H-C(NPys)); 8.67 (m, 1 H, NH-C(3')); 8.87 (m, 1 H, H-C(NPys)); 9.05 (s, 1 H, HC=N-C(6)). ^{13}C NMR (300 MHz, $CDCl_3$): δ 14.09 ($N(CH_2CH_2CH_2CH_3)$); 14.34 ($N(CH_2CH_2CH_2CH_3)$); 20.16 ($N(CH_2CH_2CH_2CH_3)$); 20.61 ($N(CH_2CH_2CH_2CH_3)$); 24.69 ($C(CH_3)_2$); 27.05 ($C(CH_3)_2$); 29.66 ($N(CH_2CH_2CH_2CH_3)$); 31.40 ($N(CH_2CH_2CH_2CH_3)$); 45.65 ($N(CH_2CH_2CH_2CH_3)$); 47.61 (C(9, Fmoc)); 52.37 (C(3')); 52.74 (C(ß, Pen)); 53.71 ($N(CH_2CH_2CH_2CH_3)$); 55.62 (2 × O-CH_3 (DMT)); 61.95 (C(α, Pen)); 63.88 (C(5')); 67.20 H_2C-O (Fmoc)); 75.06 (C(2')); 83.22; 86.94 (C(4')); 92.04 (C(1')); 113.61 (C(ar)); 120.42 (C(ar)); 121.77; 125.36; 126.76; 127.27; 127.49; 128.14; 128.27; 128.63; 130.51; 130.59; 134.62; 135.86; 136.01; 136.95 (C(8)); 141.66; 143.27; 143.96; 144.14; 144.86; 150.88; 152.76 (C(2)); 154.46 (C(NPys)); 156.79; 158.38; 158.93; 159.28 (HC=N-C(6)); 160.78; 170.03. ESI-MS (m/z): $[M+H]^+$ calcd for $C_{65}H_{70}N_{10}O_{10}S_2$ 1216.45; found 1215.40.

^{1}H NMR (300 MHz, $CDCl_3$):

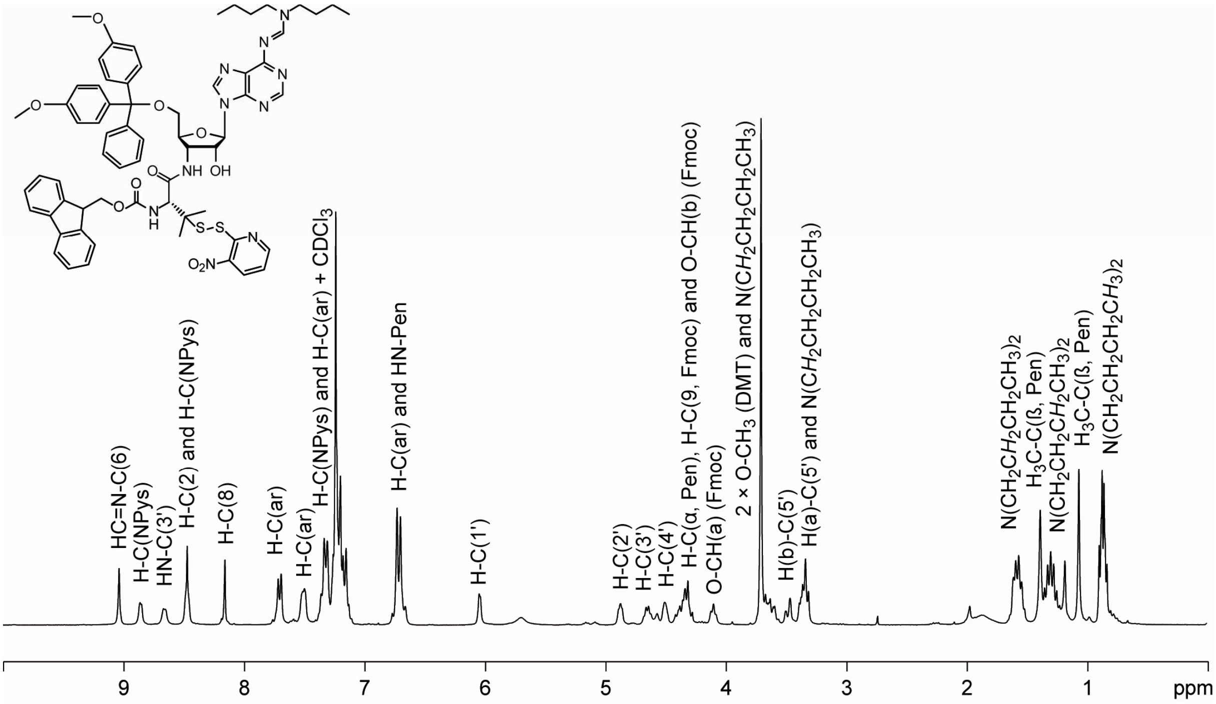

^{13}C NMR (300 MHz, $CDCl_3$):

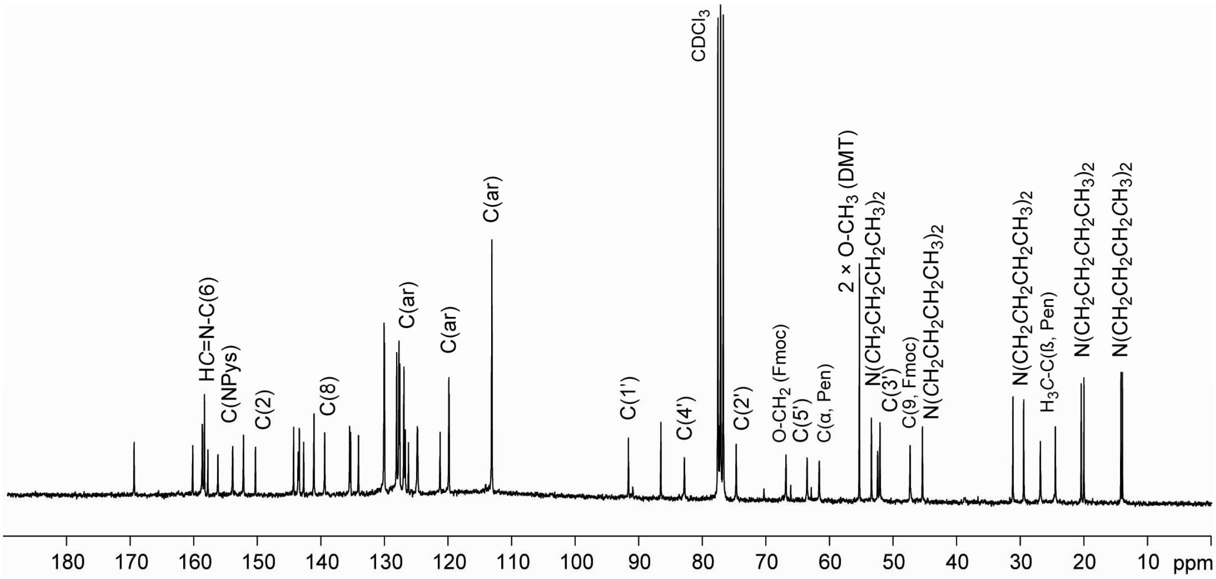

6-*N*-[(di-*n*-butylamino)methylene]-3'-{[*N*-(9-fluorenyl)methoxycarbonyl-*S*-(3-nitro-2-pyridine-sulfenyl)-L-*ß*,*ß*-dimethylcysteinyl]amino}-3'-deoxy-5'-*O*-(4,4'-dimethoxytrityl)-2'-*O*-[1,6-dioxo-6-(pentafluorophenyloxy)hexyl]-*ß*-D-adenosine (rA-Pen·2)

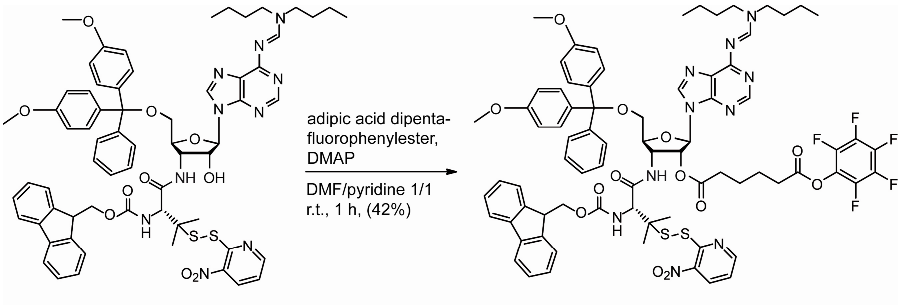

rA-Pen•1 $C_{65}H_{70}N_{10}O_{10}S_2$ [1215.44]

rA-Pen•2 $C_{77}H_{77}F_5N_{10}O_{13}S_2$ [1509.62]

Compound **rA-Pen·1** (79 mg, 0.065 mmol) was dissolved in 900 µL dry DMF and 900 µL dry pyridine. After addition of 1.1 eq 4-(dimethylamino)pyridine (DMAP, 9 mg, 0.07 mmol) and 5.5 eq adipic acid dipentafluorophenylester[5] (170 mg, 0.36 mmol) the reaction was stirred for 1 hour at room temperature under argon atmosphere. Then, the solvents were evaporated and the residue co-evaporated three times with toluene. The crude product was purified by column chromatography on SiO_2 (CH_2Cl_2/acetone, 95/5 – 80/20 v/v). The product was obtained as yellow foam.

Yield: 42 mg (0.027 mmol, 42%). TLC (CH_2Cl_2/acetone, 95/5 v/v): R_f = 0.48. ^{1}H NMR (600 MHz, $CDCl_3$): δ 0.86 – 0.95 (m, 6 H, N(CH$_2$CH$_2$CH$_2$C*H*$_3$)$_2$); 1.21 – 1.37 (m, 13 H, C(CH$_3$)$_3$ and N(CH$_2$CH$_2$C*H*$_2$CH$_3$)$_2$); 1.60 (m, 4 H, N(CH$_2$C*H*$_2$CH$_2$CH$_3$)$_2$); 1.77 (m, 4 H, OOCCH$_2$C*H*$_2$C*H*$_2$CH$_2$COOPfp); 2.49 (m, 2 H, OOCC*H*$_2$CH$_2$CH$_2$CH$_2$COOPfp); 2.68 (m, 2 H, OOCCH$_2$CH$_2$CH$_2$C*H*$_2$COOPfp); 2.82 – 3.09 (m, 2 H, H$_2$C(ß, Cys)); 3.34 – 3.45 (m, 3 H, H(a)-C(5') and N(C*H*$_2$CH$_2$CH$_2$CH$_3$)); 3.62 (m, 1 H, H(b)-C(5')); 3.74 – 3.79 (m, 8 H, 2 × O-CH$_3$ (DMT) and N(C*H*$_2$CH$_2$CH$_2$CH$_3$)); 4.22 (m, 1 H, H-C(4')); 4.40 (m, 1 H, H-C(α, Cys)); 4.55 (m, 2 H, H$_2$C=CH-C*H*$_2$); 5.17 – 5.32 (m, 3 H, *H*$_2$C=CH-CH$_2$ and H-C(3')); 5.61 (m, 1 H, HN-Cys); 5.72 – 5.95 (m, 2 H, H$_2$C=C*H*-CH$_2$ and H-C(2')); 6.17 (m, 1 H, H-C(1')); 6.76 (d, ^3J = 8.6, 4 H, H-C(ar)); 6.99 – 7.29 (m, 7 H, H-C(ar)); 7.38 (d, ^3J = 7.1, 2 H, H-C(ar)); 8.11 (s, 1 H, H-C(8)); 8.49 (s, 1 H, H-C(2)); 8.98 (s, 1 H, HC=N-C(6)). ^{13}C NMR (600 MHz, $CDCl_3$): δ 13.68 (N(CH$_2$CH$_2$CH$_2$*C*H$_3$)); 13.89 (N(CH$_2$CH$_2$CH$_2$*C*H$_3$)); 19.78 (N(CH$_2$CH$_2$*C*H$_2$CH$_3$)); 20.19 (N(CH$_2$CH$_2$*C*H$_2$CH$_3$)); 23.60 (OOCCH$_2$*C*H$_2$CH$_2$CH$_2$COOPfp); 23.92 (OOCCH$_2$CH$_2$*C*H$_2$CH$_2$COOPfp); 24.83 (C(*C*H$_3$)$_2$); 28.07 (C(*C*H$_3$)$_2$); 29.26 (N(CH$_2$*C*H$_2$CH$_2$CH$_3$)); 31.00 (N(CH$_2$*C*H$_2$CH$_2$CH$_3$)); 32.73 (OOC*C*H$_2$CH$_2$CH$_2$CH$_2$COOPfp); 33.19 (OOCCH$_2$CH$_2$CH$_2$*C*H$_2$COOPfp); 45.21 (N(*C*H$_2$CH$_2$CH$_2$CH$_3$)); 47.29 (C(9, Fmoc)); 50.70 (C(3')); 51.87 (C(ß, Pen)); 52.47 (N(*C*H$_2$CH$_2$CH$_2$CH$_3$)); 55.18 (2 × O-CH$_3$ (DMT)); 62.40 (C(α, Pen)); 63.43 (C(5')); 66.45 H$_2$C-O (Fmoc)); 75.03 (C(2')); 82.43 (C(4')); 86.66 (C(1')); 87.33; 113.18 (C(ar)); 120.03 (C(ar)); 121.26; 124.59; 124.76; 126.09; 127.11; 127.20; 127.84; 127.87; 128.21; 129.03; 130.13; 130.18; 134.13 (C(NPys)); 135.34; 135.56; 139.78 (C(8)); 141.23; 141.32; 143.11; 143.47; 143.52; 144.36; 151.31; 153.03 (C(2)); 156.78; 158.37; 158.53 (H*C*=N-C(6)); 160.14; 168.99; 169.59; 171.42. ESI-MS (m/z): $[M+H]^+$ calcd for $C_{77}H_{77}F_5N_{10}O_{13}S_2$ 1510.63; found 1509.41.

^{1}H NMR (600 MHz, $CDCl_3$):

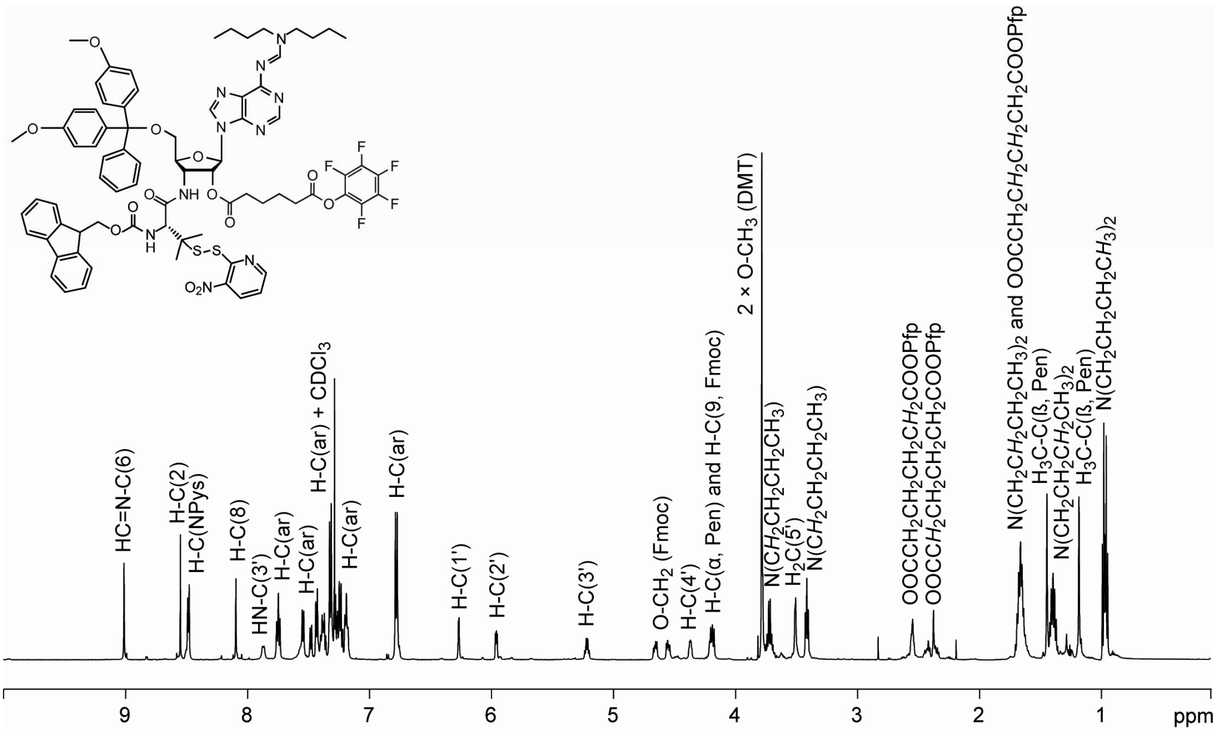

^{13}C NMR (600 MHz, $CDCl_3$):

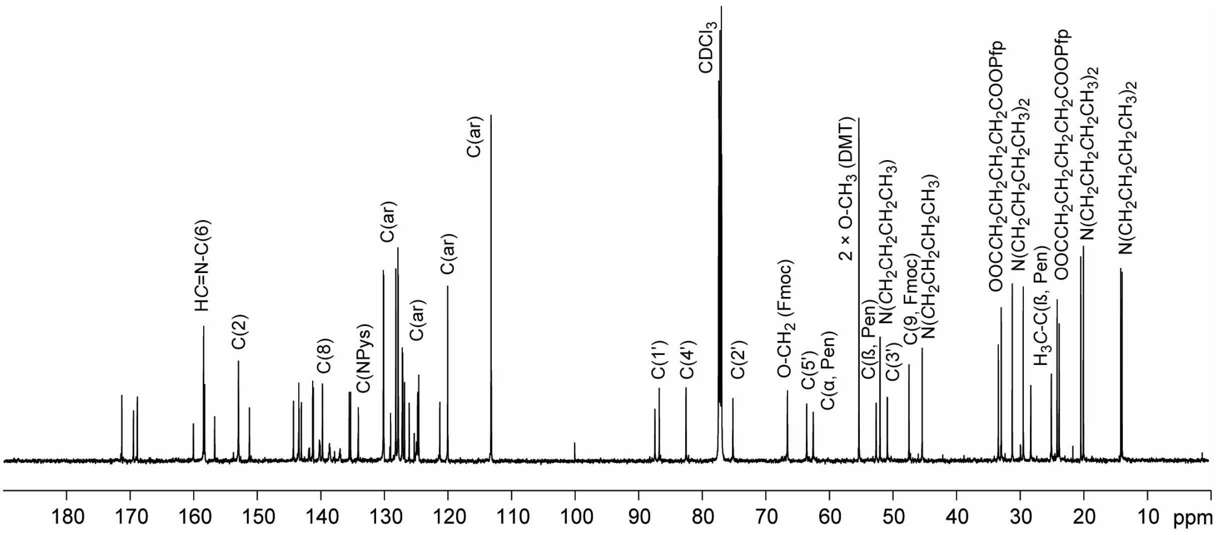

rA$^{3'NH}$-Pen solid support (rA-Pen·3)

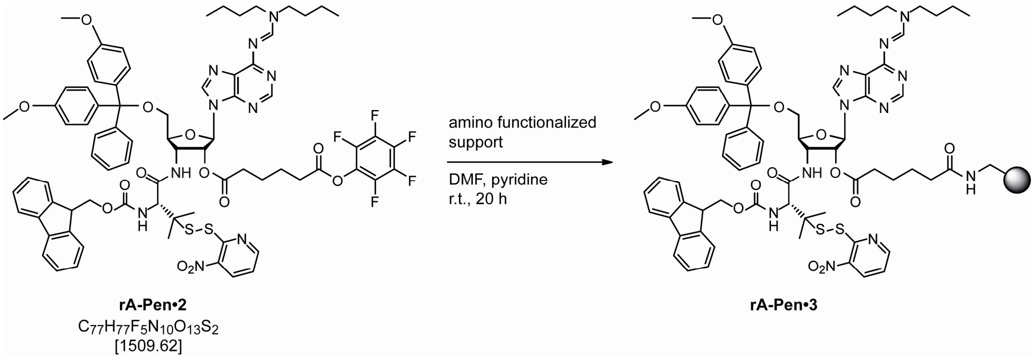

Amino-functionalized support (GE Healthcare, Custom Primer Support™ 200 Amino, 123 mg) was suspended in 700 µL dry DMF and pyridine (4 µL, 0.05 mmol). Then **rA-Pen·2** (41 mg, 0.027 mmol) was added and the suspension was agitated for 20 hours at room temperature under argon atmosphere. The beads were collected on a Büchner funnel, washed with DMF, MeOH and CH_2Cl_2 and dried under vacuum. Capping was performed by treatment with a mixture of 3 mL acetic anhydride (Ac_2O)/sym-collidine/acetonitrile (2/5/3) and 3 mL 4-(dimethylamino)pyridine (DMAP) in acetonitrile (0.5 M) for 6 minutes. After filtration the beads were washed with acetonitrile, MeOH, and CH_2Cl_2 and dried under vacuum. Loading of the support **rA-Pen·3** was 26 *µ*mol/g.

3 Synthesis of cysteine functionalized dA$^{3'NH}$ solid support dA-GlyCys

N-Allyloxycarbonyl-L-glycine

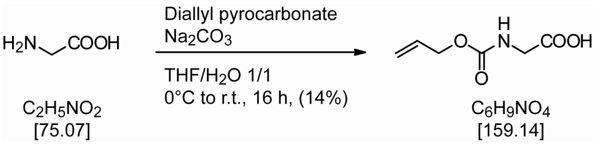

L-Glycine (1 g, 13.3 mmol) and 5.5 eq Na_2CO_3 (7.8 g, 73.3 mmol) were suspended in 60 mL tetrahydrofuran (THF)/H_2O (1/2) and cooled to 0℃. Then 1.2 eq diallyl pyro carbonate (2.6 mL, 16.0 mmol) were added and the reaction mixture stirred over night at room temperature. After washing the reaction mixture three times with diethyl ether, the aqueous phase was acidified with concentrated HCl to pH 1 and extracted three times with CH_2Cl_2. The organic phase was dried over Na_2SO_4. The product was obtained as colorless oil.

Yield: 300 mg (1.89 mmol, 14%). ^{1}H NMR (300 MHz, DMSO): δ 3.65 (d, ^3J = 6, 2 H ($H_2C(\alpha)$)); 4.48 (d, ^3J = 5, 2 H, H_2C=CH-CH$_2$); 5.16 – 5.32 (m, 2 H, H_2C=CH-CH_2); 5.90 (m, 1 H, H_2C=CH-CH$_2$); 7.48 (t, ^3J = 5, 1 H, HN); 12.52 (bs, 1 H, COOH).

^1H NMR (300 MHz, DMSO):

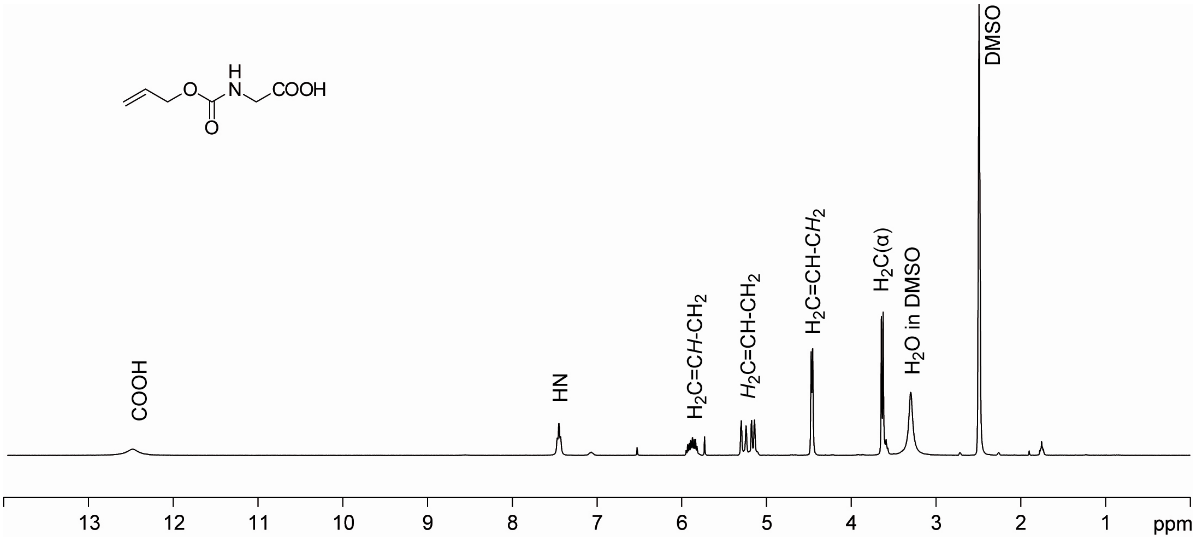

N-Allyloxycarbonyl-L-glycine pentafluorophenylester

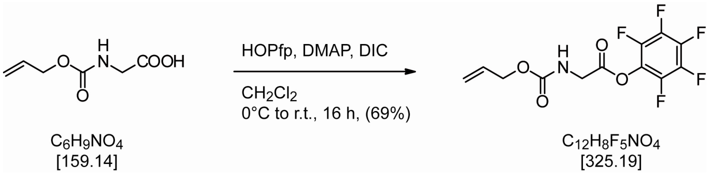

N-Allyloxycarbonyl-L-glycine (2.7 g, 17.0 mmol) was dissolved in 80 mL dry CH_2Cl_2 and cooled to 0℃. Then 1.1 eq pentafluorophenole (HOPfp, 4.4 g, 18.7 mmol), 0.2 eq 4-(dimethylamino)pyridine (DMAP, 414 mg, 3.4 mmol) and 1.2 eq *N*,*N*'-diisopropylcarbodiimide (DIC, 3.2 mL, 20.4 mmol) were added. The reaction mixture was stirred over night at room temperature. After the addition of 100 mL CH_2Cl_2, the organic phase was washed with 10% HCl, half saturated $NaHCO_3$ solution and saturated NaCl solution. The organic phase was dried over Na_2SO_4. The product was obtained as yellow oil.

Yield: 3.8 g (11.7 mmol, 69%). ^1H NMR (300 MHz, DMSO): δ 4.26 (d, ^3J = 6, 2 H ($H_2C(\alpha)$); 4.53 (m, 2 H, H_2C=CH-CH_2); 5.17 – 5.32 (m, 2 H, H_2C=CH-CH_2); 5.90 (m, 1 H, H_2C=C*H*-CH_2); 7.94 (m, 1 H, HN.

^{1}H NMR (300 MHz, DMSO):

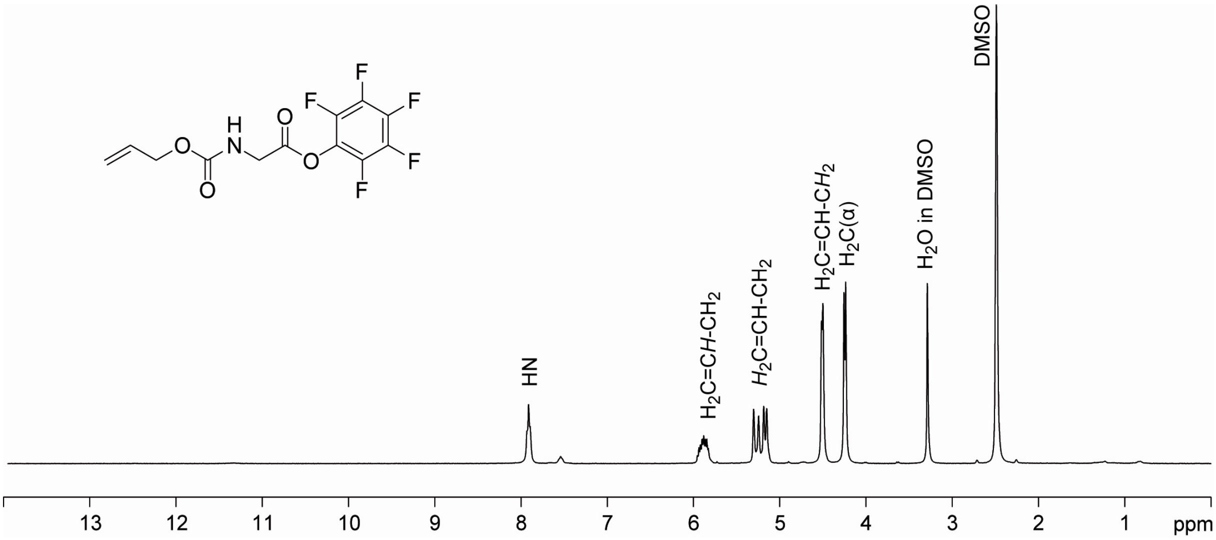

3'-*N*-[*N*-Allyloxycarbonyl-*S*-(*tert.*-butylthio)-L-glycinyl]-5'-*O*-(*tert.*-butyldimethylsilyl)-9-(3'-amino-2',3'-dideoxy-*ß*-D-ribofuranosyl)adenine (dA-Gly·1)

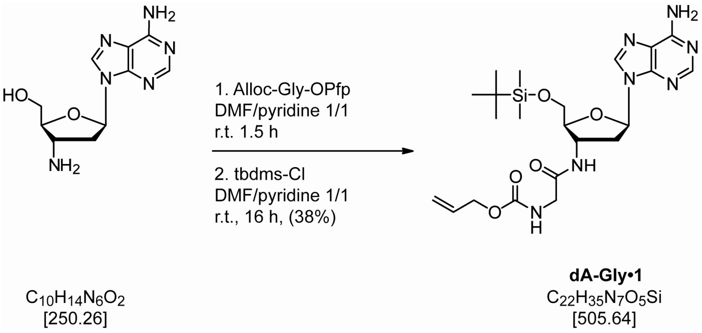

9-(3'-Amino-2',3'-dideoxy-*ß*-D-ribofuranosyl)adenine (976 mg, 3.90 mmol) and 1.1 eq *N*-allyloxycarbonyl-L-glycine pentafluorophenylester (1.4 g, 4.30 mmol) were suspended in a mixture of dry DMF (6 mL) and dry pyridine (6 mL). The reaction was stirred for 1.5 hours at room temperature under argon atmosphere. Then 1.6 eq *tert.*-butyl(chloro)dimethylsilane (941 mg, 6.24 mmol) were added and the reaction mixture was stirred for 16 hours at room temperature under argon atmosphere. The reaction was quenched with 7 mL MeOH and stirred for another 20 minutes. After evaporation of the solvents, the crude product was taken up in CH_2Cl_2 and washed with 5% citric acid, saturated $NaHCO_3$ solution, and saturated NaCl solution. The organic layer was dried over Na_2SO_4. The crude product was purified by column chromatography on SiO_2, (CH_2Cl_2/MeOH, 99/1 – 99/5 v/v). The product was obtained as colorless foam.

Yield: 746 mg (1.50 mmol, 38%). TLC (CH_2Cl_2/MeOH, 99/1 v/v): R_f = 0.56. ^{1}H NMR (300 MHz, DMSO): δ -0.01 (s, 6 H, $(CH_3)_2Si$); 0.84 (s, 9 H, $(CH_3)_3CSi$); 2.41 (m, 1 H, H(a)-C(2')); 2.71 (m, 1 H, H(b)-C(2')); 3.61 (d, ^3J = 5.9, 2 H, H_2C(α, Gly)); 3.71 (m, 1 H, H(a)-C(5')); 3.86 (m, 1 H, H-C(4')); 3.92 (m, 1 H, H(b)-C(5')); 4.48 (d, ^3J = 5.0, 2 H, H_2C=CH-CH_2); 4.56 (m, 1H, H-C(3')); 5.23 (m, 2 H,

H_2C=CH-CH$_2$); 5.91 (m, 1 H, H$_2$C=C*H*-CH$_2$); 6.38 (t, ^3J = 6.2, 1 H, H-C(1')); 7.26 (s, 2 H, H$_2$N); 7.37 (t, ^3J = 6.2, 1 H, HN-Gly); 8.14 (s, 1 H, H-C(8)); 8.29 (s, 1 H, H-C(2)); 8.31 (s, 1 H, HN-C(3')).

^{1}H NMR (300 MHz, DMSO):

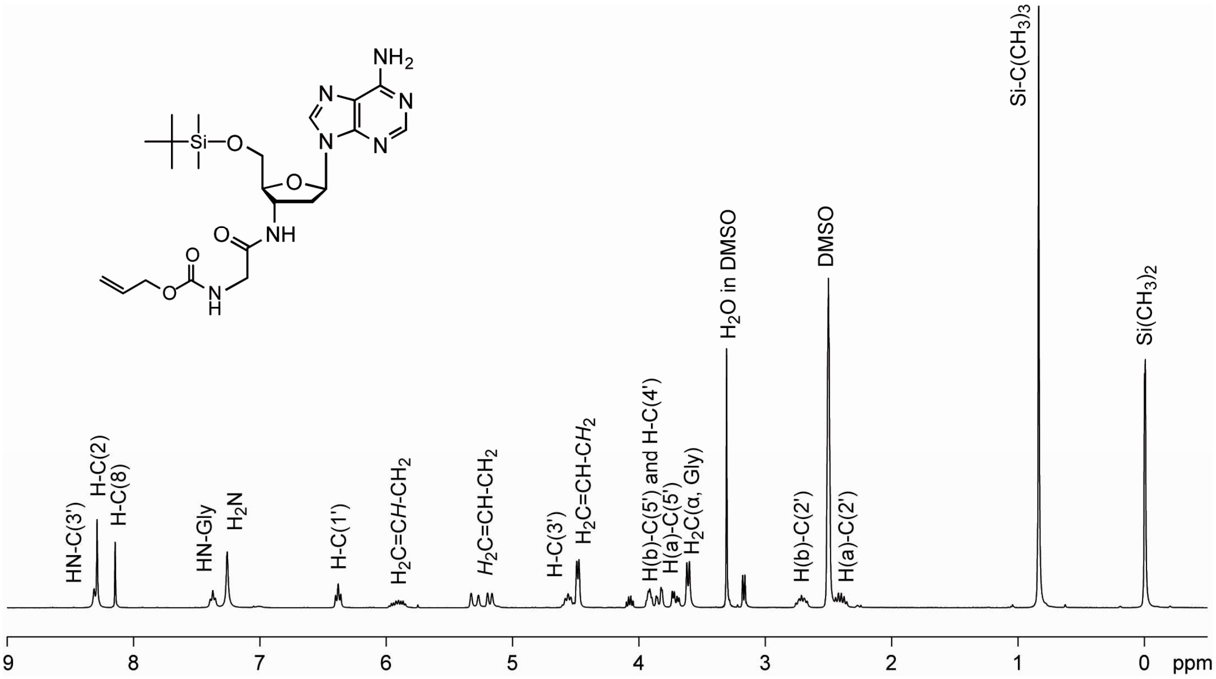

3'-*N*-[*N*-Allyloxycarbonyl-L-glycinyl]-5'-*O*-(*tert.*-butyldimethylsilyl)-*N*^{6},*N*^{6}-{2-[3-(2-carboxyethyl)] glutaryl}-9-(3'-amino-2',3'-dideoxy-*ß*-D-ribofuranosyl)adenine (dA-Gly·2)

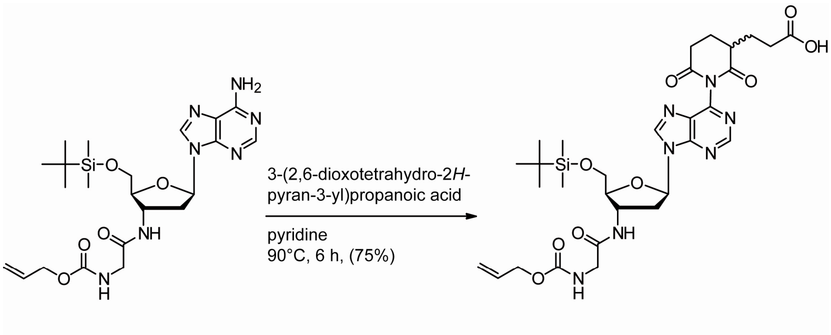

dA-Gly•1 $C_{22}H_{35}N_7O_5Si$ [505.64]

dA-Gly•2 $C_{30}H_{43}N_7O_9Si$ [673.79]

Compound **dA-Gly·1** (701 mg, 1.40 mmol) and 7.5 eq 3-(2,6-dioxotetrahydro-2*H*-pyran-3-yl)propanoic acid[6] (1.96 g, 10.5 mmol) were dissolved in 8 mL dry pyridine and stirred at 90℃ under argon atmosphere for 6 hours. After evaporation of the solvent, the residue was taken up in CH_2Cl_2 and washed three times with 5% citric acid. The organic phase was dried over Na_2SO_4 and evaporated under vacuum. The product was obtained as light brown foam.

Yield: 703 mg (1.04 mmol, 75%). TLC (CH_2Cl_2/MeOH, 99/1 v/v): R_f = 0.59. ^{1}H NMR (300 MHz, DMSO): δ -0.03 (s, 6 H, (CH$_3$)$_2$Si); 0.81 (s, 9 H, (CH$_3$)$_3$CSi); 1.75 – 2.97 (m, 9 H, linker); 2.40 (m, 1 H, H(a)-C(2')); 2.89 (m, 1 H, H(b)-C(2')); 3.65 (m, 2 H, H$_2$C(α, Gly)); 3.78 (m, 1 H, H(a)-C(5')); 3.83 (m, 1 H, H-C(4')); 3.98 (m, 1 H, H(b)-C(5')); 4.49 (d, ^3J = 5.5, 2 H, H$_2$C=CH-C*H*$_2$); 4.63 (m, 1H, H-C(3')); 5.24

(m, 2 H, H_2C=CH-CH_2); 5.91 (m, 1 H, H_2C=C*H*-CH_2); 6.55 (t, ^3J = 5.9, 1 H, H-C(1')); 7.39 (t, ^3J = 5.9, 1 H, HN-Gly); 8.34 (d, ^3J = 6.8, 1 H, HN-C(3')); 8.74 (s, 1 H, H-C(8)); 8.97 (s, 1 H, H-C(2)); 12.13 (bs, 1 H, COOH).

^{1}H NMR (300 MHz, DMSO):

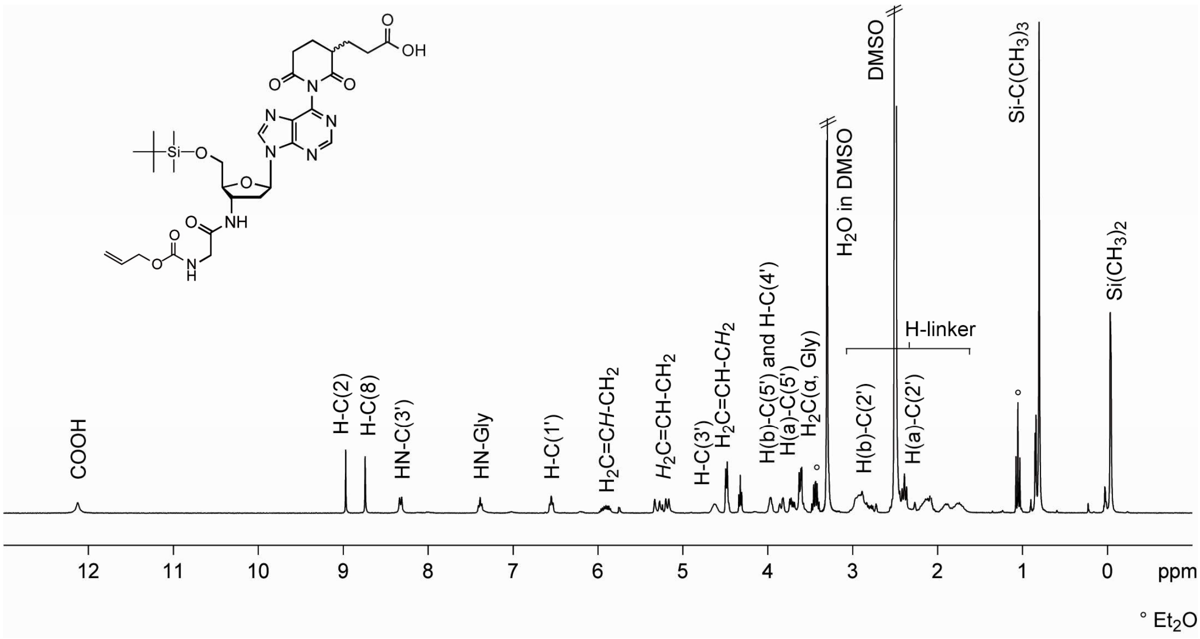

3'-*N*-[*N*-Allyloxycarbonyl-L-glycinyl]-5'-*O*-(*tert.*-butyldimethylsilyl)-*N*^{6},*N*^{6}-{2-[3-oxo-3(pentafluoro-phenyloxy)propyl]}glutaryl-9-(3'-amino-2',3'-dideoxy-*ß*-D-ribofuranosyl)adenine (dA-Gly·3)

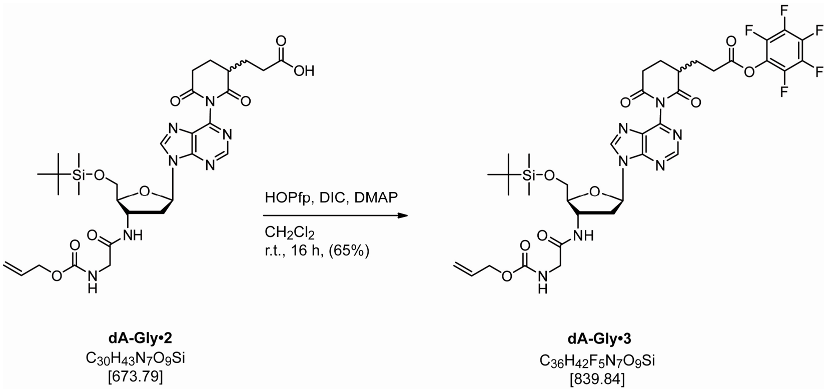

Compound **dA-Gly·2** (660 mg, 0.98 mmol) was dissolved in 16 mL dry CH_2Cl_2 and subsequently 1.1 eq pentafluorophenole (HOPfp, 198 mg, 1.08 mmol), 1.2 eq *N*,*N'*-diisopropylcarbodiimide (DIC, 182 µL, 1.18 mmol) and 0.2 eq 4-dimethylaminopyridine (DMAP, 24 mg, 0.20 mmol) were added. The reaction mixture was stirred at room temperature under argon atmosphere for 16 hours. The reaction mixture was diluted with CH_2Cl_2 and washed with 10% HCl, half-saturated $NaHCO_3$ solution and saturated NaCl solution. After drying the organic phase over Na_2SO_4, the crude product was purified by column chromatography on SiO_2 (CH_2Cl_2/acetone, 9/1 – 6/4 v/v). The product was obtained as light brown foam.

Yield: 539 mg (0.64 mmol, 65%). TLC (CH_2Cl_2/acetone, 8/2 v/v): R_f = 0.49. ^{1}H NMR (300 MHz, DMSO): δ -0.04 (s, 6 H, $(CH_3)_2Si$); 0.80 (s, 9 H, $(CH_3)_3CSi$); 1.92 – 2.30 (m, 3 H, linker); 2.54 (m, 1 H, H(a)-C(2')); 2.87 – 3.10 (m, 7 H, linker and H(b)-C(2')); 3.62 (d, 2 H, ^3J = 6.0, H_2C(α, Gly)); 3.72 (m, 1 H, H(a)-C(5')); 3.84 (m, 1 H, H(b)-C(5')); 3.97 (m, 1 H, H-C(4')); 4.89 (d, ^3J = 5.0, 2 H, $H_2C=CH-CH_2$); 4.64 (m, 1H, H-C(3')); 5.24 (m, 2 H, $H_2C=CH-CH_2$); 5.91 (m, 1 H, $H_2C=CH-CH_2$); 6.56 (t, ^3J = 5.8, 1 H, H-C(1')); 7.43 (t, ^3J = 6.0, 1 H, HN-Gly); 8.35 (d, ^3J = 7.4, 1 H, HN-C(3')); 8.76 (s, 1 H, H-C(8)); 8.99 (s, 1 H, H-C(2)). ^{13}C NMR (300 MHz, DMSO): δ -5.59 ($(CH_3)_2Si$); 13.95; 18.28 ($(CH_3)_3CSi$); 22.53; 23.29 ($(H_3C)_2HC-N=C=N-CH(CH_3)_2$); 25.36 (linker); 25.82 ($(CH_3)_3CSi$); 29.19 (linker); 29.66 ($(CH_3)_3CS$); 30.57 (linker); 32.12 (linker); 38.79 (C(2')); 40.92 (linker); 41.99 (linker); 42.88 (C(ß, Cys)); 48.19 ($(CH_3)_3CS$); 50.26 (C(α, Cys)); 54.47 (C(3')); 63.42 (C(5')); 66.00 ($H_2C=CH-CH_2$); 84.68 (C(1')); 85.87 (C(4')); 117.75 ($H_2C=CH-CH_2$); 131.08; 132.33 ($H_2C=CH-CH_2$); 136.05 (C-F); 137.67 (C-F); 139.24 (C-F); 141.02 (C-F); 142.68 (C-F); 144.41 (C(8)); 147.69; 152.28; 152.97; 155.95 (C(2)); 157.30; 168.87; 170.14; 171.27; 173.50. ESI-MS (m/z): $[M+Na]^+$ calcd for $C_{41}H_{52}F_5N_7O_9S_2Si$ 862.26; found 862.34.

^{1}H NMR (300 MHz, DMSO):

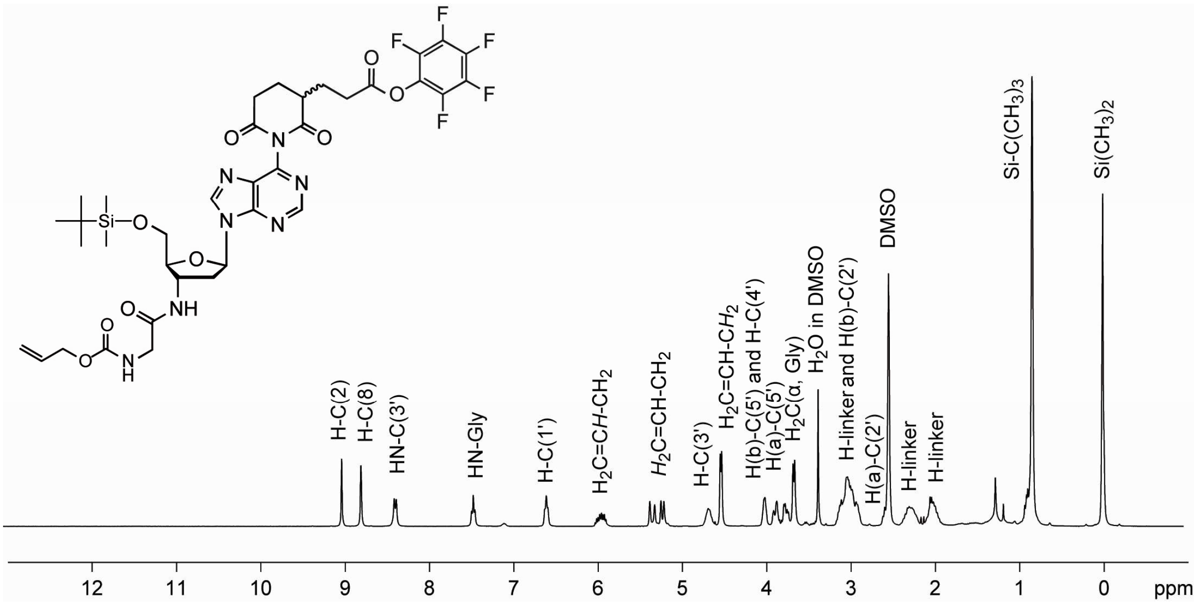

^{13}C NMR (300 MHz, DMSO):

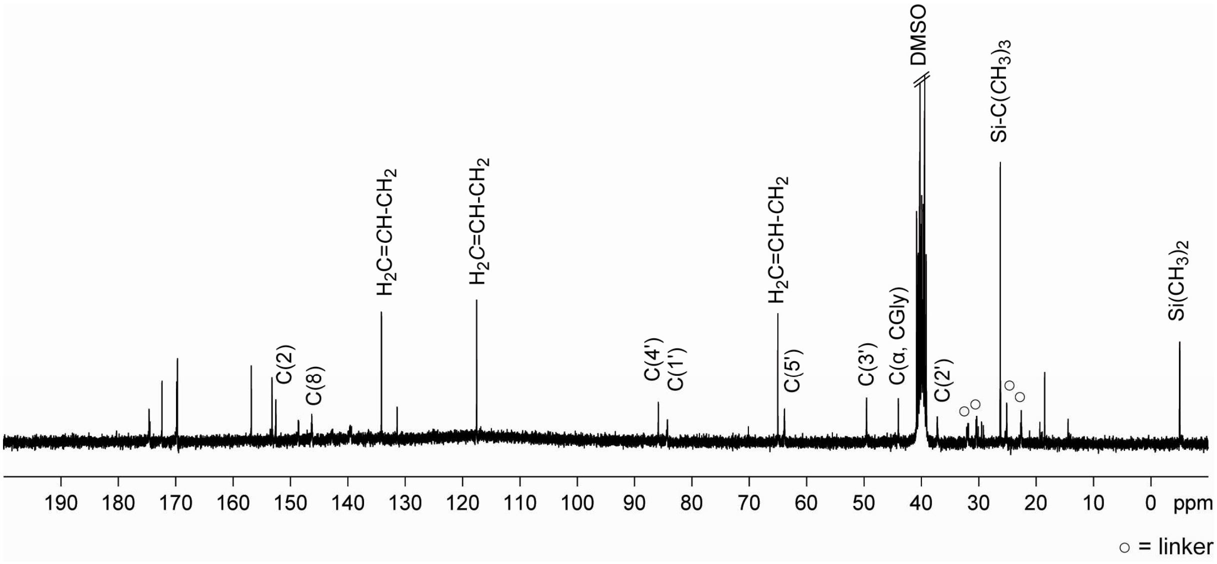

dA[3'NH]-Gly solid support (dA-Gly·4)

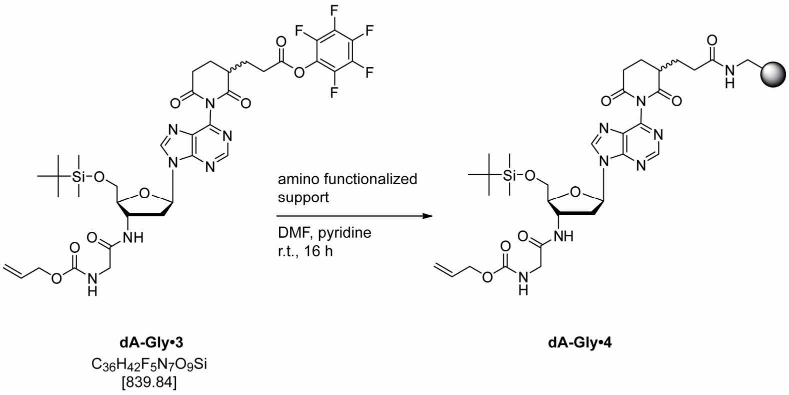

Amino-functionalized support (*GE Healthcare*, Custom Primer Support™ 200 Amino, 810 mg) was suspended in 6 mL dry DMF and pyridine (103 µL, 1.28 mmol). Then, **dA-Gly·3** (539 mg, 0.64 mmol) was added and the suspension was agitated for 16 hours at room temperature under argon atmosphere. The beads were collected on a Büchner funnel, washed with DMF, MeOH, and CH_2Cl_2 and dried under vacuum. Capping was performed by treatment with a mixture of 3 mL acetic anhydride (Ac_2O)/sym-collidine/acetonitrile (2/5/3) and 3 mL 4-(dimethylamino)pyridine (DMAP) in acetonitrile (0.5 M) for 6 minutes. After filtration, the beads were washed with acetonitrile, MeOH and CH_2Cl_2 and dried under vacuum.

dA[3'NH]-GlyCys(S*t*Bu) solid support (dA-GlyCys)

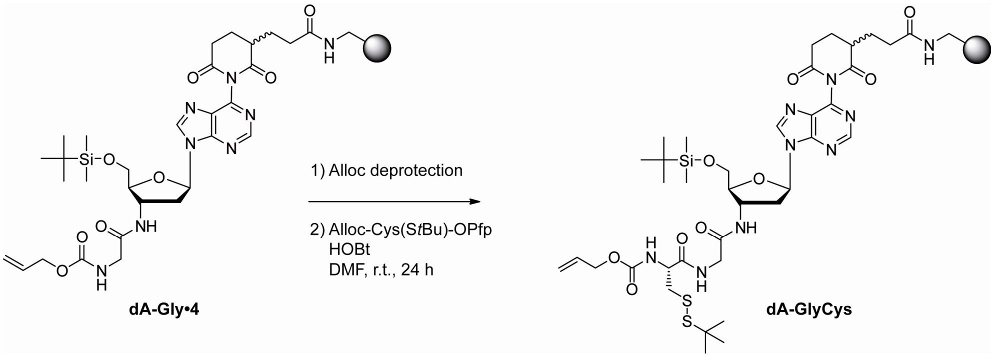

First, the solid support (100 mg) was suspended in 2.4 mL dry CH_2Cl_2 and dimethylamine borane ($(H_3C)_2NH{\cdot}BH_3$, 22.5 mg, 0.39 mmol) was added. After 10 minutes, tetrakis(triphenyl-phosphine)palladium ($(Ph_3P)_4Pd$, 75 mg, 0.06 mmol) was added. The reaction mixture was agitated for 2 hours at room temperature under argon atmosphere. The beads were collected on a small Büchner funnel, washed with 0.5 % sodium diethyldithiocarbamate hydrate in DMF (4 × 2 mL) to remove excess palladium, DMF (4 × 2 mL), MeOH (4 × 2 mL), and CH_2Cl_2 (4 × 2 mL) and dried under vacuum. Then, alloc-Cys(StBu)-OPfp (synthesized according to reference 4, 315 mg, 0.69 mmol) and hydroxybenzotriazole monohydrate (HOBt, 105 mg, 0.69 mmol) in 1.2 mL dry DMF were added to the solid support and agitated for 24 hours under argon atmosphere at room temperature. The beads were collected on a small Büchner funnel, washed with DMF (4 × 2 mL), MeOH (4 × 2 mL), and CH_2Cl_2 (4 × 2 mL) and dried under vacuum. Capping (2 × 3 min) was performed using A: Ac_2O/sym-collidine/acetonitrile (2/3/5), B: 4-(dimethylamino)pyridine in acetonitrile (0.5 M), A/B = 1/1. After capping, the beads were collected on a small Büchner funnel, washed with acetonitrile (4 × 2 mL) and CH_2Cl_2 (4 × 2 mL) and dried under vacuum.

4 Solid phase synthesis, deprotection, and purification of 3'-aminoacyl-oligonucleotides

RNA-Cys(StBu) conjugates 22 nt rA-3'-NH-Cys(StBu), 22 nt dA-3'-NH-Cys(StBu) and 18 nt rA-3'-NH-Cys(StBu) were prepared as described in reference 4.

4.1 Deprotection of the 5'-O-tert.-butyldimethylsilyl (tbdms) group of solid supports dA-Cys·4 and dA-GlyCys prior to DNA synthesis

The solid support (~30 mg) was treated with a solution of 1 M tetrabutylammonium fluoride trihydrate (TBAF·3 H_2O) and 0.5 M acidic acid in THF (1 mL) for 30 minutes. Then, the beads were collected on a small Büchner funnel, washed with DMF (4 × 2 mL), MeOH (4 × 2 mL) and CH_2Cl_2 (4 × 2 mL), and dried under vacuum.

4.2 Oligonucleotide solid phase synthesis on 3'-aminoacyl-functionalized solid supports

2'-*O*-[(Triisopropylsilyl)oxy]methyl (TOM) standard nucleoside phosphoramidites were obtained from *ChemGenes*. Oligonucleotides were synthesized on an ABI 392 Nucleic Acid Synthesizer following standard synthesis protocols (1 µmol synthesis scale). Detritylation (80 sec): dichloroacetic acid/1,2-dichloroethane (4/96); coupling (2.0 min): solutions of phosphoramidite in acetonitrile (0.1 M, 130 µL) were activated with benzylthiotetrazole in acetonitrile (0.3 M, 360 µL); capping (3 × 0.4 min): A:

Ac_2O/sym-collidine/acetonitrile (2/3/5), B: 4-(dimethylamino)pyridine in acetonitrile (0.5 M), A/B = 1/1; oxidation (1.0 min): I_2 (10 mM) in THF/pyridine/H_2O (35/10/5). Solutions of amidites, benzylthiotetrazole, and acetonitrile were dried over activated molecular sieves (4 Å) overnight. All sequences were synthesized trityl-OFF.

4.3 *Deprotection of the N-*9-(fluorenyl)methoxycarbonyl *(Fmoc) group after RNA synthesis on solid support rA-Pen·3*

After RNA synthesis, the solid support was rinsed with 20 mL of 20% piperidine in acetonitrile to remove Fmoc and cyanoethyl groups followed by washing with 20 mL acetonitrile.

4.4 Deprotection of the N-allyloxycarbonyl (alloc) group after oligonucleotide synthesis on solid supports rA-Cys·3, dA-Cys·4, and dA-GlyCys

The solid support (~30 mg) was suspended in 0.5 mL dry CH_2Cl_2 and dimethylamine borane ($(H_3C)_2NH{\cdot}BH_3$, 7.5 mg, 0.13 mmol) was added. After 10 minutes, tetrakis(triphenyl-phosphine)palladium ($(Ph_3P)_4Pd$, 25 mg, 0.02 mmol) was added. The reaction mixture was agitated for 2 hours at room temperature under argon atmosphere. Then, the beads were collected on a small Büchner funnel, washed with 0.5 % sodium diethyldithiocarbamate hydrate in DMF (4 × 2 mL) to remove excess palladium, DMF (4 × 2 mL), MeOH (4 × 2 mL), and CH_2Cl_2 (4 × 2 mL) and dried under vacuum.

4.5 Deprotection and cleavage of RNA-Pen(NPys) conjugates

3'-Penicillaminyl-RNA was deprotected with CH_3NH_2 in EtOH (8 M, 0.5 mL) and NH_3 in H_2O (32%, 0.5 mL) for 5 hours at 30℃. After filtration and evaporation to dryness, the 2'-*O*-TOM protecting groups were removed by treatment with tetrabutylammonium fluoride trihydrate in THF/H_2O (9/1) (1 M, 1.0 mL) for 5 hours at room temperature. The reaction was quenched by the addition of triethylammonium acetate pH 6.4 (1 M, 1 mL). After evaporation of THF, the solution was loaded on a HiPrep™ 26/10 desalting column (2.6 x 10 cm; Sephadex G25; *GE Healthcare*). The crude 3'-penicillaminyl-RNA was eluted with H_2O, evaporated to dryness, and dissolved in 1 mL of nanopure H_2O. During deprotection and cleavage the 3-nitro-2-pyridinesulfenyl (Npys) group of penicillamine was partly cleaved. Treatment with tris(carboxyethyl)phosphine (TCEP, 1 hour, 37℃) did not improve yields of deprotected product.

4.6 Deprotection and cleavage of a 5 nt RNA-Cys(StBu) conjugate

The 5 nt 3'-cysteinyl-RNA was deprotected with CH_3NH_2 in EtOH (8 M, 0.25 mL) and CH_3NH_2 in H_2O (40%, 0.25 mL) for 3 hours at room temperature.[7] After filtration and evaporation to dryness, the 2'-*O*-TOM protecting groups were removed by treatment with tetrabutylammonium fluoride trihydrate in THF/H_2O (9/1) (1 M, 0.5 mL) for 5 hours at room temperature. The reaction was quenched by the addition of triethylammonium acetate pH 6.4 (1 M, 0.5 mL). After evaporation of THF, the solution was loaded on a HiPrep™ 26/10 desalting column (2.6 x 10 cm; Sephadex G25; *GE Healthcare*). The crude 3'-cysteinyl-RNA was eluted with H_2O, evaporated to dryness, and dissolved in 1 mL of nanopure H_2O.

4.7 Deprotection and cleavage of DNA-Cys(StBu) conjugates

After DNA synthesis, the solid support was rinsed with 20 mL of 20% piperidine in acetonitrile followed by 20 mL acetonitrile to remove cyanoethyl groups. 3'-Cysteinyl-DNA was deprotected with NH_3 in H_2O (32%, 0.6 mL) and EtOH (0.2 mL) for 16 hours at 55°C. After filtration and evaporation to dryness, the crude 3'-cysteinyl-DNA was dissolved in 1 mL of nanopure H_2O.

4.8 Purification of 3'-aminoacyl-oligonucleotides

Analysis of 18 nt and 22 nt crude 3'-aminoacyl-oligonucleotides after deprotection was performed by anion-exchange chromatography on a *Dionex* DNAPac® PA-100 column (4 × 250 mm). Flow rate: 1 mL/min; eluant A: 25 mM Tris·HCl (pH 8.0), 6 M urea; eluant B: 25 mM Tris·HCl (pH 8.0), 6 M urea, 500 mM $NaClO_4$; 60°C; gradient: 0-60% B in A within 40 min; UV-detection at 260 nm. For the 5 nt crude 3'-aminoacyl-oligonucleotide the gradient 0-40% B in A within 30 min was used. Crude products were purified using optimized gradients on a semipreparative *Dionex* DNAPac® PA-100 column (9 × 250 mm), flow rate: 2 mL/min. Fractions containing the product were loaded on a C18 SepPak®Plus cartridge (*Waters*/*Millipore*), washed with 0.1 M triethylammonium carbonate buffer, H_2O, and eluted with H_2O/acetonitrile (1/1). Combined fractions of the 3'-aminoacyl-oligonucleotides were lyophilized to dryness. The purified 3'-penicillaminyloligonucleotides were isolated as homodisulfides.

4.9 Mass spectrometry of 3'-aminoacyl-oligonucleotides

The purified 3'-aminoacyl-oligonucleotides were characterized by mass spectrometry on a *Finnigan* LCQ Advantage MAX ion trap instrumentation connected to an *Amersham* Ettan micro LC system (negative-ion mode with a potential of -4 kV applied to the spray needle). LC: Sample: 200 pmol of 3'-penicillaminyloligonucleotide dissolved in 30 µL of 20 mM ethylenediaminetetraacetic acid (EDTA) solution; average injection volume: 30 µL; column (Xbridge®MS, C18 2.5 µm; 1.0 × 50 mm) at 21°C; flow rate: 30 µL/min; eluant A: 8.6 mM triethylamine, 100 mM 1,1,1,3,3,3-hexafluoro-2-propanol in H_2O (pH 8.0); eluant B: MeOH; gradient: 0-100% B in A within 20 min; UV detection at 254 nm. The 5 nt purified 3'-aminoacyl-oligonucleotide was analyzed without the addition of EDTA.

5 Preparation of amino-modified peptide thioester MRFF-ABT

5.1 Solid phase peptide synthesis

The peptide was synthesized manually in a 2 mL syringe with a filter membrane following standard Fmoc/*t*Bu synthesis protocols. Fmoc deprotection: 20% piperidine in DMF (2 × 500 µL, 8 min and 12 min). Coupling: hydroxybenzotriazole monohydrate (HOBt, 2 eq), *O*-(benzotriazol-1-yl)-*N*,*N*,*N*',*N*'-tetramethyluronium hexafluorophosphate (HBTU, 2 eq), *N*,*N*-diisopropylethylamine (DIPEA, 4 eq), and Fmoc-protected amino acid (2 eq, 0.4 M) in DMF; two coupling cycles with each 60 minutes. Between each step the resin was washed with DMF (4 × 1 mL). After the last coupling step, the resin was washed with CH_2Cl_2 (4 × 1 mL). The peptide was synthesized with N-terminal Boc protection using Boc-Met-OH in the last coupling step.

Peptide thioesters MFFG-ABT, MLLT-ABT, MRVL-ABT, and MRVW-ABT were synthesized as described in reference 4. Leu-ABT was prepared as described in reference 9.

5.2 *Synthesis of peptide thioester MRFF-ABT*

For the synthesis of the protected peptide acid, phenylalanine-loaded 2-chlorotrityl resin (H-*L*-Phe-2-chlorotrityl resin, 200-400 mesh, loading: 0.87 mmol/g, *Iris Biotech*) was used. After manual Fmoc/*t*Bu solid phase peptide synthesis, the resin was treated with trifluoroethanol/CH_2Cl_2 (2/8, 15 mL cleavage solution per gram resin) for 45 minutes at room temperature to cleave the crude protected peptide acid.[8] The filtrate was collected and the resin was washed twice with cleavage solution and once with DMF. The united filtrates were concentrated, precipitated in a mixture of cold diethyl ether/hexane (2/1, 30 mL per gram resin), and centrifuged at 4℃ for 30 minutes (*Hermle* Z300K, 6000 rpm). The precipitate was washed twice with cold diethyl ether and then lyophilized out of acetonitrile/H_2O (1/1). The crude protected peptide acid was analyzed by RP-HPLC and ESI-MS, and used without further purification.

The ABT group was synthesized in five steps according to the literature.[9] The ABT group (1.4 eq) and the crude protected peptide acid were dissolved with 1-ethyl-3-(3-dimethylaminopropyl)carbodiimide (EDC·HCl, 4.2 eq) and 4-(dimethylamino)pyridine (DMAP, 4.2 eq) in dry CH_2Cl_2 under argon atmosphere and stirred for 2 hours at room temperature.[10] After evaporation of the solvents, the residue was taken up in ethylacetate, washed three times with 1 M HCl, three times with 10% $NaHCO_3$, and once with saturated NaCl solution. The organic phase was dried over Na_2SO_4 and evaporated. The protected peptide thioester was deprotected with trifluoroacetic acid (TFA)/triisopropylsilane (TIPS)/H_2O (88/10/2, 1.5 mL, 2 hours), precipitated in cold *tert.*-butylmethyl ether/hexane (2/1, 20 mL) and centrifuged at 4℃ fo r 30 minutes (*Hermle* Z300K, 6000 rpm). The precipitate was washed twice with cold *tert.*-butylmethyl ether/hexane (2/1) and then lyophilized out of acetonitrile/H_2O (1/1).

5.3 *Analysis and purification of peptides*

Analysis of crude products was performed by reversed phase (RP) chromatography on a *GE Healthcare* 3 mL Resource™ RPC column (6.4 × 100 mm). Flow rate: 2 mL/min; eluant A: 0.1% trifluoroacetic acid (TFA) in H_2O; eluant B: 0.08% TFA in acetonitrile; 25℃; gradi ent: 5-90% B in A within 20 column volumes; UV-detection at 210 nm. The crude product was purified on the same column using an optimized gradient. Fractions containing peptide were collected and lyophilized.

5.4 *Mass spectrometry of peptides*

Peptides were characterized by electrospray ionization (ESI) mass spectrometry on a *Finnigan* LCQ Advantage MAX ion trap instrumentation connected to an *Amersham* Ettan micro LC system (positive-ion mode with a potential of +4 kV applied to the spray needle). The sample was solubilized in acetonitrile/H_2O (1/1). Conditions for flow injections were 70% B with a flow rate of 100 µL/min; eluant A: 0.05% trifluoroacetic acid (TFA) in acetonitrile; eluant B: 0.05% TFA in H_2O.

6 Desulfurization of 3'-peptidyl-oligonucleotides after native chemical ligation

Native chemical ligation (NCL) was performed according to the conditions described in reference 4. After NCL of 3 nmol oligonucleotide, the reaction solution was diluted with 100 µL water and transferred into a centrifugal concentrator (Vivaspin® 500, membrane: 3000 MWCO PES, product number: VS0191) and centrifuged (Eppendorf MiniSpin®, 13400 rpm) for 20 minutes at room

temperature to desalt the reaction solution from buffer and dispose of excess peptide thioester and thiophenole. To the remaining solution in the upper reservoir of the centrifugal concentrator 500 µL 0.1 M ammonium citrate were added and the solution was centrifuged again for 20 minutes at room temperature. After a final washing step with 500 µL water, the concentrated solution in the upper reservoir of the centrifugal concentrator was lyophilized to dryness. For desulfurization, 1.2 µL sodium phosphate pH 7.5, 2 µL 0.5 M tris(carboxyethyl)phosphine (TCEP) pH 7.3, 1 µL 20 mM glutathione, 0.8 µL 100 mM 2,2'-azobis(2-methylpropionamidine)dihydrochloride (V-50) were added. Each reagent stock solution was prepared freshly and degassed prior to use. Final concentrations for desulfurization were as followed: $c_{conjugate}$ = 0.6 mM, 200 mM TCEP, 16 mM V-50, 4 mM glutathione, 240 mM sodium phosphate pH 7.5. The reaction solution was exposed to ultrasound for 10 seconds and kept under argon atmosphere. Reactions were carried out at 37°C for 6 (short conjugates) to 12 hours (longer conjugates).

6.1 Analysis and purification of 3'-peptidyl-oligonucleotides after native chemical ligation/desulfurization

An aliquot of the reaction mixture was injected for anion-exchange HPLC analysis onto a *Dionex* DNAPac® PA-100 column (4 × 250 mm). Flow rate: 1 mL/min; eluant A: 25 mM Tris·HCl (pH 8.0), 6 M urea; eluant B: 25 mM Tris·HCl (pH 8.0), 6 M urea, 500 mM $NaClO_4$; 60℃; gradient: 0-60% B in A within 40 min; UV-detection at 260 nm. For the isolation of products, the reaction mixture was diluted with 100 µL water and injected directly onto the same column, using the same conditions as described above. Fractions containing product were loaded on a C18 SepPak®Plus cartridge (*Waters*/*Millipore*), washed with 0.1 M triethylammonium carbonate, H_2O, and eluted with H_2O/acetonitrile (1/1). Combined fractions of the desulfurized 3'-peptidyl-oligonucleotides were lyophilized.

6.2 Mass Spectrometry of 3'-peptidyl-oligonucleotides

The purified 3'-peptidyl-oligonucleotides were characterized by mass spectrometry on a *Finnigan* LCQ Advantage MAX ion trap instrumentation connected to an *Amersham* Ettan micro LC system (negative-ion mode with a potential of -4 kV applied to the spray needle). LC: Sample: 200 pmol dissolved in 30 µL of 20 mM ethylenediaminetetraacetic acid (EDTA) solution; average injection volume: 30 µL; column (Xbridge®MS, C18 2.5 µm; 1.0 × 50 mm) at 21℃; flow rate: 30 µL/min; eluant A: 8.6 mM triethylamine, 100 mM 1,1,1,3,3,3-hexafluoro-2-propanol in H_2O (pH 8.0); eluant B: MeOH; gradient: 0-100% B in A within 20 min; UV detection at 254 nm. The purified 5 nt 3'-peptidyl-oligonucleotide was analyzed without the addition of EDTA.

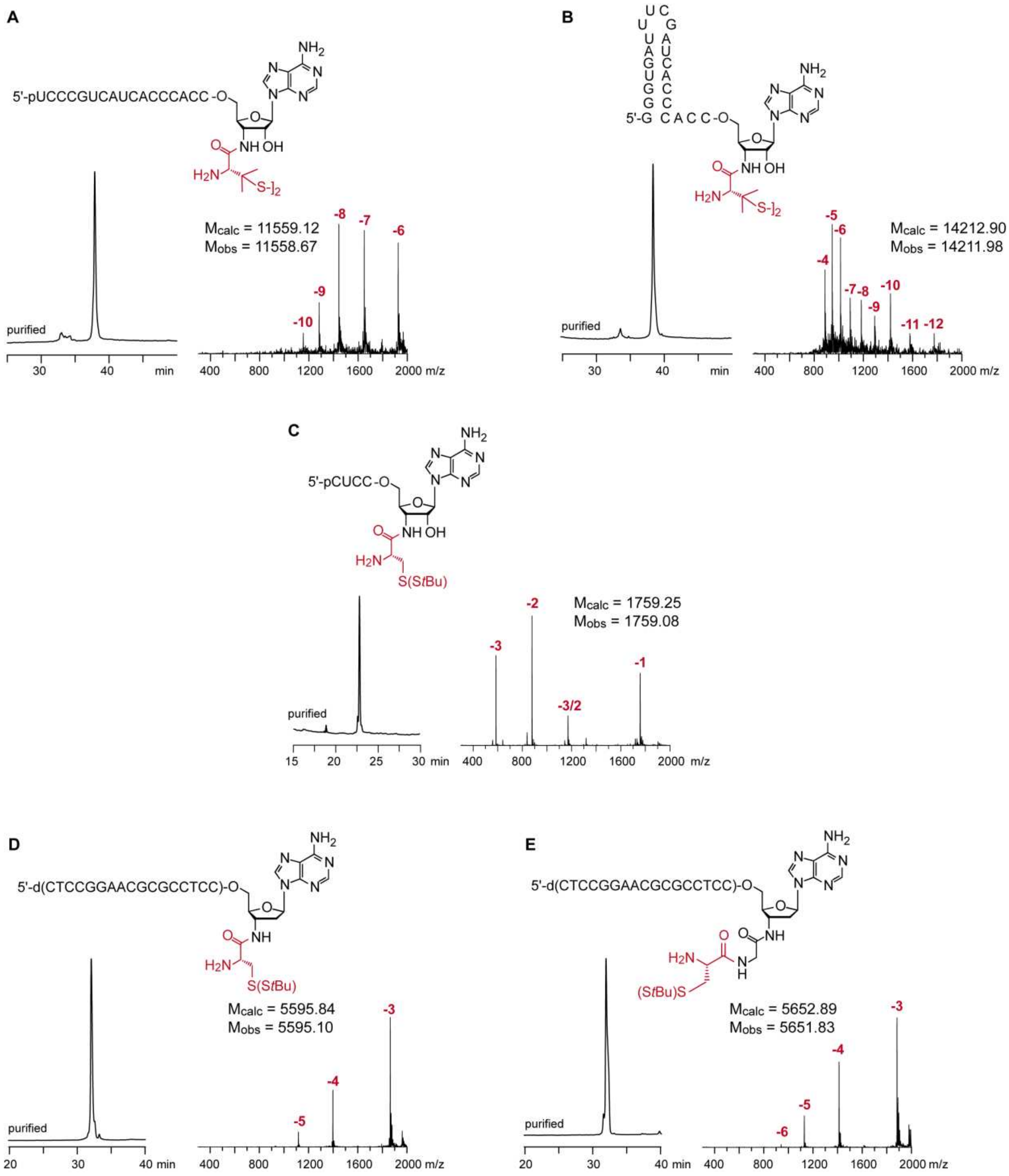

Supporting Figure 1. Analytical data of the purified 3'-aminoacyl-oligonucleotides used for NCL/desulfurization. Anion-exchange HPLC traces (left, see section 6.1 for details) and LC-ESI mass spectra (right, see section 6.2 for details) of purified 3'-penicillaminyl- (**A**, **B**) and 3'-cysteinyl-oligonucleotides (**C**, **D**, **E**).

A: 5'-pCUCCGGAACGCGCCUCCA-3'-NH-Pen-$)_2$

B: 5'-GGGUGAUUUCGAUCACCCACCA-3'-NH-Pen-$)_2$

C: 5'-pCUCCA-3'-NH-Cys(S*t*Bu)

D: 5'-d(CTCCGGAACGCGCCTCC)dA-3'-NH-Cys(S*t*Bu)

E: 5'-d(CTCCGGAACGCGCCTCC)dA-3'-NH-GlyCys(S*t*Bu)

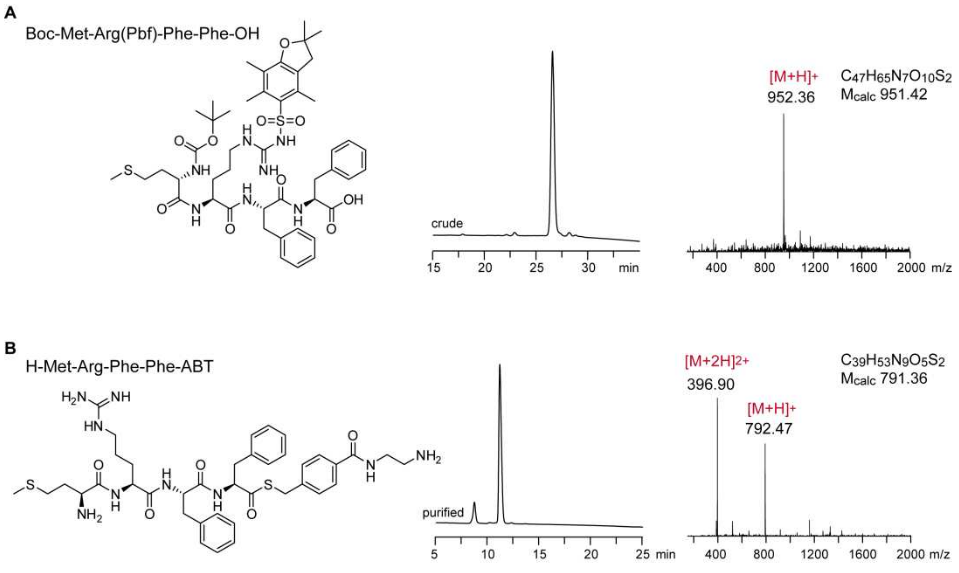

Supporting Figure 2. Analytical data of the crude protected peptide acid Boc-MR(Pbf)FF-OH (**A**) and the purified peptide thioester MRFF-ABT (**B**). Shown are RP HPLC traces (left, see section 5.3 for details) and ESI mass spectra (right, see section 5.4 for details).

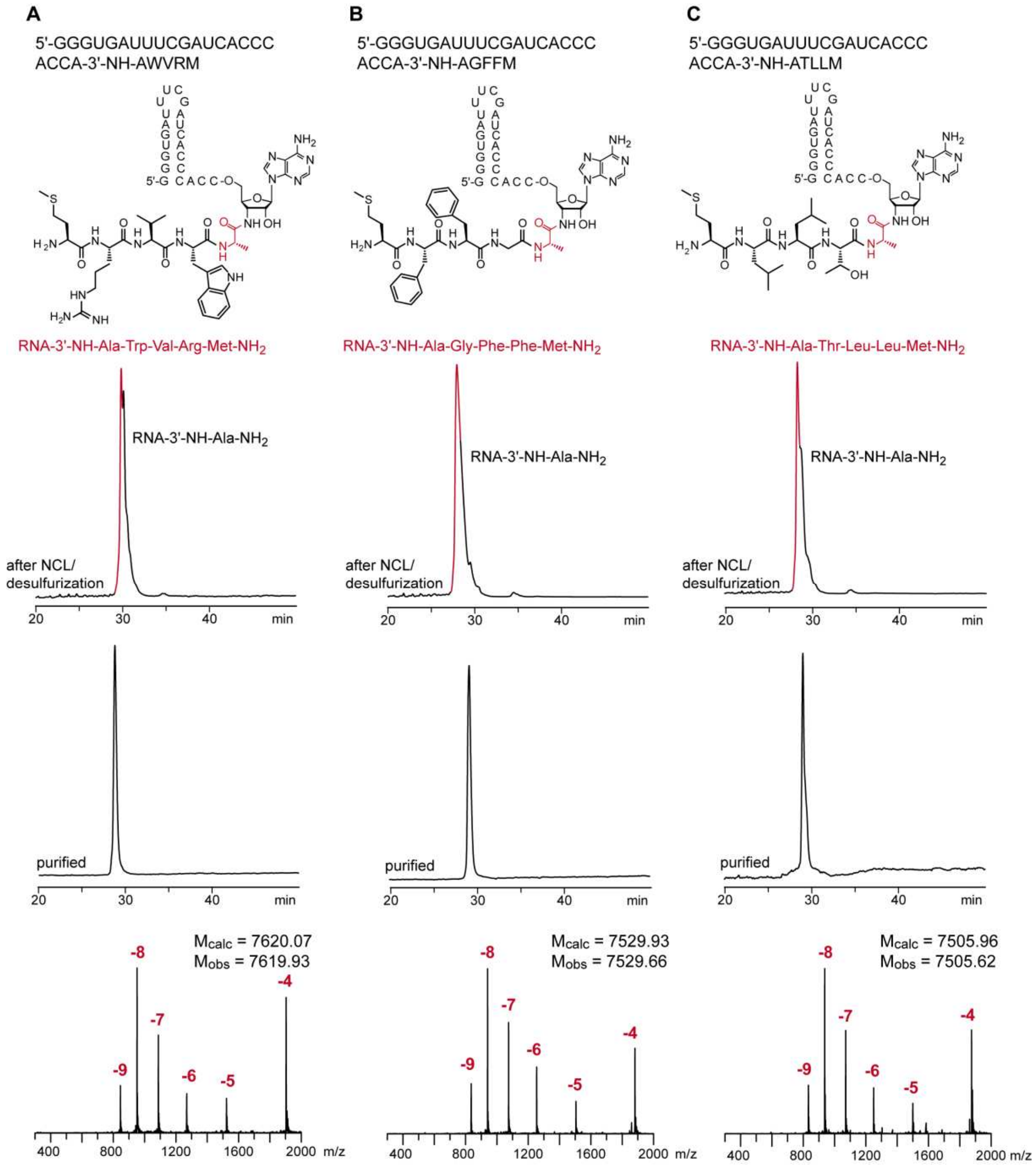

Supporting Figure 3. Alanine-containing 3'-peptidyl-tRNA mimics obtained by NCL/desulfurization. Shown are the anion-exchange HPLC traces (top, see section 6.1 for details) after NCL/desulfurization, the anion-exchange HPLC traces of the purified products (middle) and their LC-ESI mass spectra (bottom, see section 6.2 for details). Conditions for NCL: c_{RNA} = 0.25 mM, $c_{peptide\text{-}ABT}$ = 8 mM, 0.1 M TCEP, 2% (v/v) PhSH, 1 M Tris·HCl pH 8.0, 20 h, 25℃.[4] Desulfurization conditions: $c_{conjugate}$ = 0.6 mM, 200 mM TCEP, 16 mM V-50, 4 mM glutathione, 240 mM sodium phosphate pH 7.5, 12 h, 37℃ (section 6).

A: 5'-GGGUGAUUUCGAUCACCCACCA-3'-NH-AWVRM

B: 5'-GGGUGAUUUCGAUCACCCACCA-3'-NH-AGFFM

C: 5'-GGGUGAUUUCGAUCACCCACCA-3'-NH-ATLLM

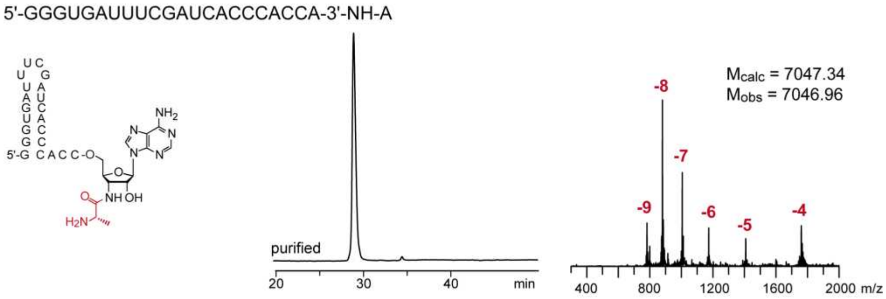

Supporting Figure 4. The 3'-alanyl-RNA conjugate 5'-GGGUGAUUUCGAUCACCCACCA-3'-NH-A was isolated as byproduct of NCL/desulfurization of 3'-cysteinyl-RNA. Shown are the anion-exchange HPLC trace (see section 6.1 for details) of the purified byproduct and the LC-ESI mass spectrum (see section 6.2 for details). Conditions for NCL: c_{RNA} = 0.25 mM, $c_{peptide\text{-}ABT}$ = 8 mM, 0.1 M TCEP, 2% (v/v) PhSH, 1 M Tris·HCl pH 8.0, 20 h, 25℃.[4] Desulfurization conditions: $c_{conjugate}$ = 0.6 mM, 200 mM TCEP, 16 mM V-50, 4 mM glutathione, 240 mM sodium phosphate pH 7.5, 12 h, 37℃ (section 6).

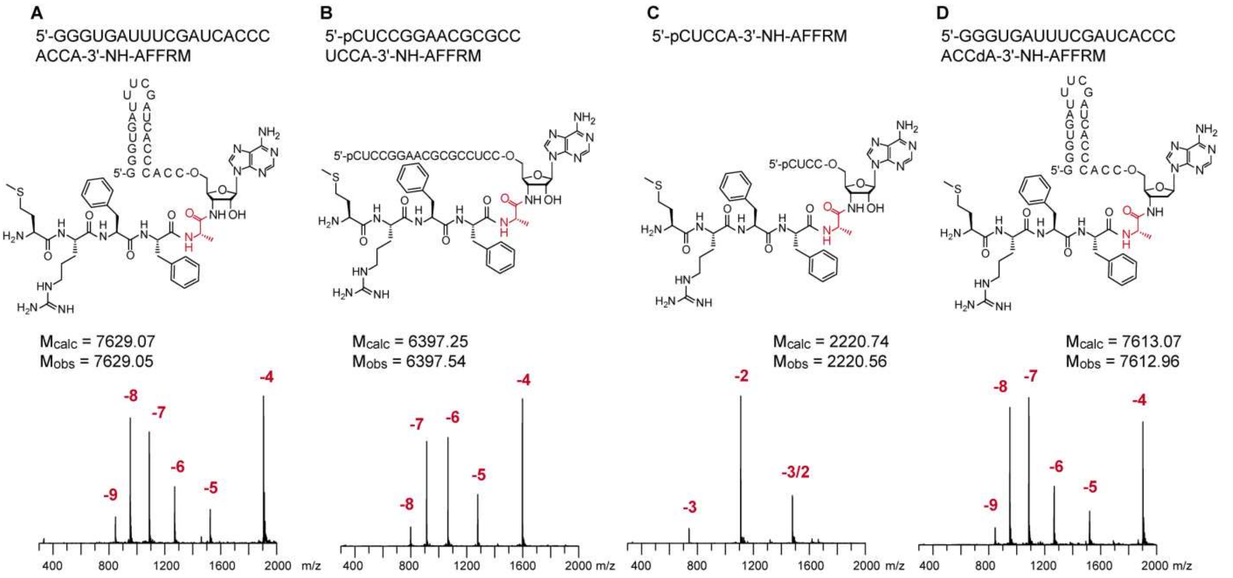

Supporting Figure 5. Series of 3'-peptidyl-tRNA mimics prepared by NCL/desulfurization whose peptide moiety MRFFA confers resistance to the macrolide antibiotic cethromycin.[11] Shown are the LC-ESI mass spectra (see section 6.2 for details) of the isolated products. Conditions for NCL with peptide thioester MRFF-ABT: c_{RNA} = 0.25 mM, $c_{MRFF\text{-}ABT}$ = 8 mM, 0.1 M TCEP, 2% (v/v) PhSH, 1 M Tris·HCl pH 8.0, 20 h, 25℃.[4] Desulfurization conditions: $c_{conjugate}$ = 0.6 mM, 200 mM TCEP, 16 mM V-50, 4 mM glutathione, 240 mM sodium phosphate pH 7.5, 12 h, 37℃ (section 6).

A: 5'-GGGUGAUUUCGAUCACCCACCA-3'-NH-AFFRM

B: 5'-pCUCCGGAACGCGCCUCCA-3'-NH-AFFRM

C: 5'-pCUCCA-3'-NH-AFFRM

D: 5'-GGGUGAUUUCGAUCACCCACCdA-3'-NH-AFFRM

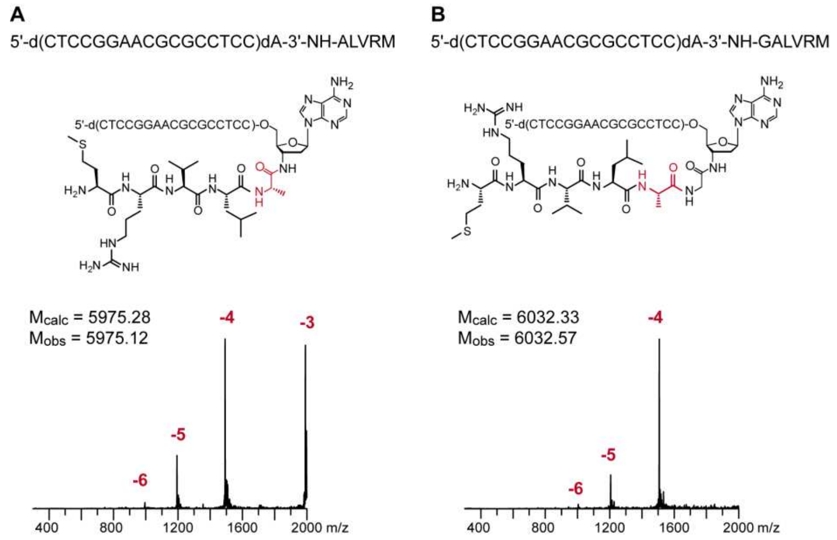

Supporting Figure 6. NCL/desulfurization of 3'-cysteinyl-DNA conjugates. Conditions for NCL with peptide thioester MRVL-ABT: c_{RNA} = 0.25 mM, $c_{MRVL\text{-}ABT}$ = 8 mM, 0.1 M TCEP, 2% (v/v) PhSH, 1 M Tris·HCl pH 8.0, 20 h, 25℃.[4] Desulfurization conditions: $c_{conjugate}$ = 0.6 mM, 200 mM TCEP, 16 mM V-50, 4 mM glutathione, 240 mM sodium phosphate pH 7.5, 12 h, 37℃ (section 6). Shown are the LC-ESI mass spectra (see section 6.2 for details) of the isolated products.

A: 5'-d(CTCCGGAACGCGCCTCC)dA-3'-NH-ALVRM

B: 5'-d(CTCCGGAACGCGCCTCC)dA-3'-NH-GALVRM

5'-GGGUGAUUUCGAUCACCCACCA-3'-NH-VGFFM

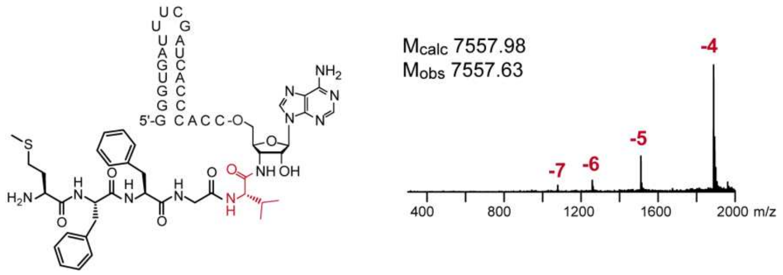

Supporting Figure 7. NCL/desulfurization of a 22 nt 3'-penicillaminyl-RNA conjugate. Conditions for NCL with peptide thioester MFFG-ABT: c_{RNA} = 0.25 mM, $c_{MFFG\text{-}ABT}$ = 8 mM, 0.1 M TCEP, 2% (v/v) PhSH, 1 M Tris·HCl pH 8.0, 20 h, 25℃.[4] Desulfurization conditions: $c_{conjugate}$ = 0.6 mM, 200 mM TCEP, 16 mM V-50, 4 mM glutathione, 240 mM sodium phosphate pH 7.5, 12 h, 37℃ (section 6). Shown is the LC-ESI mass spectrum (see section 6.2 for details) of the isolated valine-containing product peptide-RNA conjugate 5'-GGGUGAUUUCGAUCACCCACCA-3'-NH-VGFFM.

References

[1] Y.-C. J. Chen, F. Hansske, K.D. Janda, M.J. Robins *J. Org. Chem*. **1991**, *56*, 3410-3413.

[2] B.C. Froehler, M.D. Mateucci *Nucl. Acids Res*. **1983**, *11*, 8031-8036.

[3] H. Moroder, J. Steger, D. Graber, K. Fauster, K. Trappl, V. Marquez, N. Polacek, D. Wilson, R. Micura *Angew. Chem. Int. Ed.* **2009**, *48*, 4056-4060.

[4] A.-S. Geiermann, N. Polacek, R. Micura, *J. Am. Chem. Soc.* **2011**, *133*, 19068-19071.

[5] J. Steger, D. Graber, H. Moroder, A.-S. Geiermann, M. Aigner, R. Micura *Angew. Chem. Int. Ed. Engl*. **2010**, *49*, 7470-7472.

[6] W. Pfleiderer, S.R. Waldvogel *Helv. Chim. Acta* **1998**, *81*, 46-58.

[7] S. Pitsch, A. Weiss, L. Jenny, A. Stutz, X. Wu *Helv. Chim. Acta* **2001**, *84*, 3773-3795.

[8] Novabiochem, Peptide Synthesis 2010/2011 catalog, Method 3-25.

[9] N. Niwa, Y. Yamagishi, H. Murakami, H. Suga *Bioorg. Med. Chem. Lett*. **2009**, *19*, 3892-3894.

[10] Y. Goto, H. Suga *J. Am. Chem. Soc.* **2009**, *131*, 5040-5041.

[11] V. Vimberg, L. Xiong, M. Bailey, T. Tenson, A. Mankin *Mol. Microbiol.* **2004**, *54*, 376-385.
